# Recent advances in availability and synthesis of the economic costs of biological invasions

**DOI:** 10.1093/biosci/biad060

**Published:** 2023-08-22

**Authors:** Danish A Ahmed, Phillip J Haubrock, Ross N Cuthbert, Alok Bang, Ismael Soto, Paride Balzani, Ali Serhan Tarkan, Rafael L Macêdo, Laís Carneiro, Thomas W Bodey, Francisco J Oficialdegui, Pierre Courtois, Melina Kourantidou, Elena Angulo, Gustavo Heringer, David Renault, Anna J Turbelin, Emma J Hudgins, Chunlong Liu, Showkat A Gojery, Ugo Arbieu, Christophe Diagne, Boris Leroy, Elizabeta Briski, Corey J A Bradshaw, Franck Courchamp

**Affiliations:** Center for Applied Mathematics and Bioinformatics, Department of Mathematics and Natural Sciences, Gulf University for Science and Technology, Hawally, Kuwait; Center for Applied Mathematics and Bioinformatics, Department of Mathematics and Natural Sciences, Gulf University for Science and Technology, Hawally, Kuwait; Department of River Ecology and Conservation, Senckenberg Research Institute and Natural History Museum Frankfurt,Gelnhausen, Germany; South Bohemian Research Center of Aquaculture and Biodiversity of Hydrocenoses, Faculty of Fisheries and Protection of Waters, University of South Bohemia in České Budějovice, Vodňany, Czech Republic; Institute for Global Food Security, School of Biological Sciences at Queen's University Belfast, Belfast, NorthernIreland; School of Arts and Sciences at Azim Premji University, Bangalore, India; School of Arts and Sciences, Azim Premji University, Bhopal, India; Society for Ecology, Evolution, and Development, Wardha, India; South Bohemian Research Center of Aquaculture and Biodiversity of Hydrocenoses, Faculty of Fisheries and Protection of Waters, University of South Bohemia in České Budějovice, Vodňany, Czech Republic; South Bohemian Research Center of Aquaculture and Biodiversity of Hydrocenoses, Faculty of Fisheries and Protection of Waters, University of South Bohemia in České Budějovice, Vodňany, Czech Republic; Department of Basic Sciences in the Faculty of Fisheries at Muğla Sıtkı Koçman University, in Muğla, Turkey; Department of Life and Environmental Sciences in the Faculty of Science and Technology at Bournemouth University, Poole, Dorset, England, United Kingdom; Graduate Program in Conservation and Ecotourism at the Federal University of Rio de Janeiro State, Rio de Janeiro, Rio de Janeiro State, Brazil; Institute of Biology at Freie Universität Berlin, Berlin, Germany; Neotropical Limnology Group, at the Federal University of Rio de Janeiro State, Rio de Janeiro, Rio de Janeiro State, Brasil; Laboratório de Ecologia e Conservação in the Departamento de Engenharia Ambiental, Setor de Tecnologia, at the Universidade Federal do Paraná, in Curitiba, Paraná, Brazil; School of Biological Sciences at King's College, University of Aberdeen, Aberdeen, Scotland, United Kingdom; South Bohemian Research Center of Aquaculture and Biodiversity of Hydrocenoses, Faculty of Fisheries and Protection of Waters, University of South Bohemia in České Budějovice, Vodňany, Czech Republic; Centre for Environmental Economics—Montpellier, National Institute for Research in Agriculture and the Environment, Montpellier, France; Department of Sociology, Environmental and Business Economics, University of Southern Denmark, Esbjerg Ø, Denmark; Université de Bretagne Occidentale, Plouzané, France; Estación Biológica de Doñana, Seville, Spain; Departamento de Ecologia e Conservação in the Instituto de Ciências Naturais at the Universidade Federal de Lavras, Lavras, Minas Gerais, Brazil; Nürtingen-Geislingen University, Nürtingen, Germany; Centre National de Recherche Scientifique's Ecosystèmes, Biodiversité, Evolution, University of Rennes, Rennes, France; Université Paris–Saclay, CNRS, AgroParisTech, Ecologie Systématique Evolution, Gif-sur-Yvette, France; Great Lakes Forestry Centre at Canadian Forestry Services, part of Natural Resources Canada, Sault Ste Marie, Ontario, Canada; Department of Biology at Carleton University, Ottawa, Ontario, Canada; College of Fisheries at the Ocean University of China, Qingdao, China; Institute of Hydrobiology at the Chinese Academy of Sciences, Wuhan, China; Department of Botany at the University of Kashmir, Kashmir, India; Université Paris–Saclay, CNRS, AgroParisTech, Ecologie Systématique Evolution, Gif-sur-Yvette, France; Senckenberg Biodiversity and Climate Research Centre, Frankfurt am Main, Germany; Smithsonian Conservation Biology Institute, at the National Zoological Park, Front Royal, Virginia, United States; Centre de Biologie pour la Gestion des Populations, at Institut de Recherche pour le Développement, Montferrier-sur-Lez Cedex, France; Unité Biologie des Organismes et des Ecosystèmes Aquatiques, Muséum National d’Histoire Naturelle, Sorbonne Universités, Université de Caen Normandie, Université des Antilles, in Paris, France; GEOMAR Helmholtz-Zentrum für Ozeanforschung Kiel, Kiel, Germany; Global Ecology Laboratory, Partuyarta Ngadluku Wardli Kuu, College of Science and Engineering, Flinders University, Adelaide, South Australia; ARC Centre of Excellence for Australian Biodiversity and Heritage, Wollongong, New South Wales, Australia; Université Paris–Saclay, CNRS, AgroParisTech, Ecologie Systématique Evolution, Gif-sur-Yvette, France

**Keywords:** environmental management, guiding policy, InvaCost, invasive alien species, economic impacts

## Abstract

Biological invasions are a global challenge that has received insufficient attention. Recently available cost syntheses have provided policy- and decision makers with reliable and up-to-date information on the economic impacts of biological invasions, aiming to motivate effective management. The resultant *InvaCost* database is now publicly and freely accessible and enables rapid extraction of monetary cost information. This has facilitated knowledge sharing, developed a more integrated and multidisciplinary network of researchers, and forged multidisciplinary collaborations among diverse organizations and stakeholders. Over 50 scientific publications so far have used the database and have provided detailed assessments of invasion costs across geographic, taxonomic, and spatiotemporal scales. These studies have provided important information that can guide future policy and legislative decisions on the management of biological invasions while simultaneously attracting public and media attention. We provide an overview of the improved availability, reliability, standardization, and defragmentation of monetary costs; discuss how this has enhanced invasion science as a discipline; and outline directions for future development.

The globalization of economies and societies has accelerated trade, travel, and connectivity, increasing the potential for exchanges of biological material between distant regions and eroding natural biogeographical barriers (Vander Zanden and Olden 2008, Duffy et al. 2017). Increased global trade and transport have concomitantly accelerated the translocation of alien species to unprecedented rates (Essl et al. 2011, Seebens et al. 2017). The intentional (e.g., sectors of primary production such as agriculture or leisure activities) or unintentional (e.g., by hitchhiking) introductions of alien species by humans can result in the establishment of self-sustaining populations that consequently spread within their new environment (i.e., invasive alien species; hereafter, *invasive species* for brevity), with the potential to cause staggering ecological (Blackburn et al. 2011, Bellard et al. 2021), health (Zhang et al. 2022), social (Bacher et al. 2018), and economic damages (Bradshaw et al. 2016, Diagne et al. 2021a).

Biological invasions are among the main drivers of global biodiversity loss through their degradation of ecological communities and functions (Bellard et al. 2022); of alteration of functional diversity (Milardi et al. 2019, Kaushik et al. 2022, Renault et al. 2022b); of disruption of ecosystem services such as pollination, freshwater provisioning, nutrient cycling, and soil fertility (Pejchar and Mooney 2009, Vilà et al. 2010); and of massive economic losses worldwide (Bradshaw et al. 2016, Diagne et al. 2021a). This has propelled invasion biology as a major discipline in ecology, and a resulting greater awareness of their threats and rates of introduction highlights the need to prevent, control, and eradicate invasive species (Simberloff et al. 2013).

However, biological invasions have arguably not yet been recognized to the extent the problems they impose deserve, either by the public or by policymakers (Courchamp et al. 2017). Unawareness arising in part from the diversity and complexity of ecological impacts and resulting insufficient communication of the problems caused by biological invasions are at least partly responsible for this lack of recognition (Courchamp et al. 2017). Moreover, even with the baseline knowledge that biological invasions are mostly problematic, the severity of the problem is widely underestimated. Using economic costs as a proxy is part of the solution to promote understanding, awareness, and support of biological invasions as a major problem (Diagne et al. 2020a). In fact, biological invasions have reached a similar magnitude of costs to other major natural hazards (Turbelin et al. 2023). The neglect of the importance and risks conveyed by invasions is accentuated through doubt cast by works questioning the severity of the problem (see Russell and Blackburn 2017 for a review).

In addition, challenges such as unclear definitions, a lack of general rules, a multitude of impact- and risk-assessment scoring systems, inadequate funding, and inappropriate or absent policies have reduced awareness and uptake of management approaches for biological invasions in conservation programs (Courchamp et al. 2017, Jarić et al. 2020). The resulting concepts, theories, natural and social threats, and laws have made it difficult to navigate the field and to disseminate novel findings, especially for decision-makers. These challenges have led to insufficient efforts to mitigate biological invasions in tandem with other major global environmental changes (e.g., climate change), resulting in large, negative impacts that potentially could have been circumvented had they been addressed earlier (Ahmed et al. 2022). It is therefore essential to derive universal and standardized approaches to communicate the scale of the problem posed by biological invasions and to motivate adequate action.

Public health and monetary costs are salient metrics to communicate the impacts of biological invasions with policymakers and the public (Diagne et al. 2021a, Zhang et al. 2022), and economic assessments represent a familiar and readily standardized measure of impact across contexts. Reliable information on the damage caused by biological invasions and the costs of managing them is also essential for setting priorities and developing effective management strategies, because the comparison of management expenditures and damage costs can be used for the calibration and targeting of public policies (Dana et al. 2014, Booy et al. 2020). A limitation to overcome has been the lack of openly available information on the monetary costs arising from invasive species. Overarching attempts to improve governance approaches for greater proactiveness toward invasions—for example, by the European Commission (European 2014) or the United States (Executive Office of the President 2016) and Canadian (Reid et al. 2021) governments—have successfully highlighted the importance of controlling invasive species and formulated management prescriptions. However, policies often lack a strong evidence base, which weakens their operational effectiveness (Faulkner et al. 2020, Lukey and Hall 2020). The cornerstone is the compilation of priority species at the territorial level—for example, the Union list that constitutes the core of invasive species regulation 1143/2014 within the European Union. This list includes invasive species that undergo thorough risk assessment, are subjected to peer review, and are selected by consensus among the European Commission and its member states. Although the assessment methods on which these lists are based occasionally mention the economic costs caused by invasions, the lack of objectivity and broadscale cost information and the flaws and untested assumptions of many local and regional cost studies (Bradshaw et al. 2016, Diagne et al. 2021a) are major drawbacks.

Monetary evaluation comes with limitations because the attribution of monetary to nonmarket values faces methodological pitfalls (Hausman 2012, Johnston et al. 2017) but also because of biases related to the difficulty of accounting for the completeness of impacts and their measurement (Spangenberg and Settele 2010) and the subjectivity of human perceptions of their values (Shackleton et al. 2019). Assigning a monetary value to a nonmarket phenomenon such as ecosystem degradation or the extinction of an endemic species will necessarily be an imperfect proxy, so the first question is whether it is morally appropriate to use such a proxy, followed by how to use such a value and how to ensure its reliability. Reliability itself is contingent on improvements limiting methodological biases, to reveal preferences for nonmarket values, and to systematize the reporting and assessment of impacts. Methodological advances are constantly being made to reduce bias and broaden applications (Carson 2012, Freeman III et al. 2014, Rakotonarivo et al. 2016), and a growing number of studies are mobilizing these methods to assess the impacts of invasions and address reporting artefacts (Diagne et al. 2021a). As for their use, the monetary values of these damages are to be taken with caution and call for the systematization of reliability indices considering the uncertainties and incompleteness of estimates.

Evaluations of monetary impacts from biological invasions have been hampered by differences in research effort, national wealth, and the translation of environmental impacts to monetary terms (Hudgins et al. 2023). Monetary impacts are notably dynamic processes, and the effects on any one economy are subject to change as new invasive species arrive or damageable economic sectors emerge. The monetization of impacts relies on reporting and evaluation to make costs available, which means that the distribution of global costs is heavily influenced by uneven reporting, including research effort and publication bias. The magnitude of the negative impacts of invasive species on economies is also potentially influenced by cumulative wealth, because larger economies have more assets to damage (and that cost more) or potentially invest more in research that reports costs (Haubrock et al. 2021). Similarly, variation in purchasing power means that economic costs are borne differently among countries, irrespective of whether the costs themselves have been standardized to a common currency and year (Diagne et al. 2020b). Even if costs are of a lower monetary value, the effects could be more pronounced in lower-income nations that have lower preparedness and purchasing power (dx.doi.org/10.21203/rs.3.rs-2444595/v1; preprint [not peer reviewed]). Consequently, cost assessments should be viewed in the context of these limitations, which simultaneously modulate the extent of economic impact and underestimate their effects.

Although broadscale economic cost estimates had been made previously, they were often based on unsupported extrapolations, unrealistic assumptions, or undocumented sources (Pimentel et al. 2000, 2001, 2005, Kettunen et al. 2009, Pimentel 2011). To address these issues, structured and reproducible syntheses of monetary impacts from biological invasions have gained traction recently through the *InvaCost* database (Diagne et al. 2020b, Leroy et al. 2022). These recent works have shifted the focus of investigation into the monetary costs of invasive species across regions and taxonomic scales (Zenni et al. 2021) and have demonstrated how effective control can benefit legislators and stakeholders (Ahmed et al. 2022, Cuthbert et al. 2022). The InvaCost project has established a sound socioeconomic and political foundation for policy guidance—for example, by underpinning impact assessments in ongoing reports by the Intergovernmental Science-Policy Platform on Biodiversity and Ecosystem Services (https://ipbes.org) and informing European Union decision-making around proactive management investments (https://environment.ec.europa.eu/research-and-innovation/science-environment-policy_en). In the present article, we synthesize the advances made through this extended approach, highlighting knowledge gaps and identifying future research avenues. Our main objective is to provide a comprehensive overview of this process, which necessarily includes a detailed description of *InvaCost*, given its recent widespread uptake in the field of invasion costs.

## Pioneering cost-synthesis studies

Prior to the release of the *InvaCost* database, most attempts to inventory the monetary costs of invasive species at broad scales were subjective, lacked empirical support, or were forged from speculation (see also box 1). In many cases, cost estimates were given without citation, were obsolete, referred to untraceable sources such as personal communications with experts or unpublished data, or the sources cited—when cross-referenced—did not report the numbers claimed (see the incidences provided in Lovell and Stone 2005, Oreska and Aldridge 2010, Goldstein 2011). Where citations were traceable and reliable, many estimates were crude, arising from overestimation—for example, a total cost multiplied by the proportion of invasive species among many candidate species within the system or extrapolations of case studies to the entire (often only suspected) distribution or population size of a particular invasive species. In addition, many of these regional costs were based on extrapolations that drew from bioeconomic models that went beyond the intended contexts (e.g., Hoagland and Jin 2006, Yemshanov et al. 2009, Goldstein 2011, McDermott et al. 2013). Moreover, explanations for extrapolations, when provided, often had logical flaws, or the multipliers used had no empirical basis (i.e., they were not measured quantitatively; Goldstein 2011).

Box 1. **Urban legends in invasion biology.**

Perpetuating citations of retracted papers in ecology spreads misinformation and retards research (Cosentino and Veríssimo 2016). An analogous phenomenon occurs in invasion science, where citations of irreproducible estimates spread unreliable information. However, irreproducible estimates can sometimes be concealed within convoluted citation chains.

For example, reviews of the Formosan termite *Coptotermes formosanus* in peer-reviewed journals suggest this species costs nearly US${\$}$40 billion per year globally (Rust and Su 2012, Ahmad et al. 2021); many studies cite these reviews to justify this estimate. However, the price tag was an unsubstantiated and unreferenced increase on a previous estimate of US${\$}$22 billion per year (Su 2002), but even that estimate was a dubious speculation extrapolated from a single value of US${\$}$1.5 billion per year for the United States (Su 1994). Digging deeper, it turns out that the United States–wide value came from another source (Su 1991), which was itself extrapolated from an unrefereed report in a symposium proceedings that only gave an unsubstantiated estimate of US${\$}$60 million for Hawai'i in 1985.

Therefore, an irreproducible local estimate of US${\$}$60 million in 1985 eventually mushroomed—without evidence—into a global value of US${\$}$40 billion per year by 2021, gaining the unwarranted endorsement of peer review in the process. Although this is an egregious example, it is unfortunately not an isolated case. Misguided outcomes can similarly occur when extrapolating invasive species environmental impacts by simply multiplying their *per capita* effects by their abundance and range size, because impacts might not relate linearly with abundance, area occupied, or other scales (Sofaer et al. 2018).

We therefore urge invasion biologists to adopt best-citation practices (Teixeira et al. 2013, Sanz-Martín et al. 2016) to avoid unsubstantiated statements and inflation. We recommend striving to cite original cost values (i.e., where their calculation methodology is presented when referring to a specific cost), identifying when the original sources cannot be accessed or confirmed, highlighting uncertainties and inconsistencies in any dubious estimates, and providing detailed methods and justifications whenever updating or modifying previous estimates. Overall, authors should clarify where they have calculated the cost themselves (using traceable steps that allow reproducibility) or cited a primary/secondary source. The onus is therefore on cost reporters to be vigilant, detailed, and authentic. Wylie and Janssen-May (2017) is an example where robust damage cost estimates for the red imported fire ant (*Solenopsis invicta*) in Australia are provided, with all the information necessary to reproduce the calculations, original sourced references, indication of the currencies and years, and how they accounted for inflation.

To demonstrate these issues, consider the four major papers that provided cost estimates of invasive species at a global, national, or regional scale (Pimentel et al. 2000, 2001, 2005, Pimentel 2011) prior to the advent of the *InvaCost* database. These four sources, updated in succession, have consistently served as primary references in invasion cost-related studies, to date receiving several thousands of citations and underpinning other influential studies. Despite these acknowledged limitations, values from these publications are still cited today without recognition of the embedded limitations. Although the cost estimates formulated therein were arguably the best and the only available at that time and brought attention to the problem of invasions, in hindsight, they all contained serious flaws.

For example, Sagoff (2008) outlined several flaws in the cost values presented, and Holmes and colleagues (2009) identified large biases in the estimates of damages arising from forest pests. In addition, many values were not accompanied by any supporting citations or raw data, such as the value of 50,000 nonindigenous species introduced to the United States. The purported cost of zebra mussels at US${\$}$1 billion per year (US Department of State 2009) was not supported, although a credible source cited the costs of this species at substantially more than US${\$}$1 billion per year (O'Neill 1997). Another example in Pimentel (2011) cited a cost of US${\$}$631 million for West Nile virus, but the corresponding table therein (their table 1) provides no citation, making it difficult for the reader to trace the source. Other references contained costs from non-peer-reviewed sources (e.g., Armstrong 1995) that were, themselves, based on unpublished reports that were impossible to source. Still other calculations were based on unsubstantiated costs, such as those given for rats (*Rattus* spp.; without citation) as US${\$}$15 per rat per year and birds killed by cats (US${\$}$30 per bird per year; critiqued by Goldstein 2011, Lamb 2013). Similarly, neither the data nor calculations were summarized in Pimentel and colleagues (2001) for the values of introduced mammals to Brazil (Mares et al. 1989). Other calculations were based on flawed logic—the total cost of dog bites was given as US${\$}$250 million per year, when most bites are from domestic (pet) dogs and not invasive (stray) dogs. Weeds were reported (US Bureau of the Census 1998) to cause a 12% loss in yield, which translated to US${\$}$32 billion per year. However, this was based on the erroneous assumption that the damage by weed species to crops are distributed equally; a similar line of reasoning was applied to crop insect pests (US${\$}$14.4 billion per year), forest insect pests (US${\$}$2.1 billion per year), plant pathogens for crops (US${\$}$21.5 billion per year), and for forests (US${\$}$2.1 billion per year). On the basis of an unsubstantiated damage cost of US${\$}$200 per pig per year, they implicitly assumed that every pig (*Sus scrofa*) attacks crops and therefore entails this per-capita cost. This was the same approach taken to estimate the damage arising from starlings (*Sturnus* spp.; US${\$}$800 million per year).

Perhaps the most egregious examples of unsubstantiated estimates concerns the reported costs for fire ants (*Solenopsis* spp.) and Formosan termites (*Coptotermes formosanus*; see also box 1). The *Solenopsis* estimates were derived from dubious sources (from a newspaper article, Corn et al. 2002; irretrievable, Vinson 1992) and then simply multiplied by the number of US states where the species were present, to arrive at a total value of US${\$}$1 billion per year. This figure has been cited many times since and continues to garner citations. Other estimates were grounded in ecological *non sequuntur* (identified by Goldstein 2011). For example, if rats (that eat grain and other human commodities) are deemed to cost US${\$}$19 billion, and cats (that eat birds) are deemed to cost US${\$}$17 billion, but the beneficial impact of cats eating rats is ignored, these values are an overestimate. The papers also confused control and damage costs (identified by Sagoff 2008). The estimated control cost of one pigeon in a localized survey was multiplied by an unsubstantiated estimate of the total number of pigeons, resulting in valuing the cost to control all pigeons (if such a program existed). But this cost was instead claimed to be the cost of the total accrued damage caused by them.

Building on and improving these pioneer works, the next generation of global assessments of economic damages due to invasions were mostly focused on few model taxa (Bradshaw et al. 2016), specific economic sectors (Paini et al. 2016) or ecosystems (Lovell et al. 2006), and limited spatial scales (Hoffmann and Broadhurst 2016). Despite their potential shortcomings, most published studies had the benefit of raising awareness about the consequences associated with the monetary burden of invasive species. Moreover, although it is being essential for the purpose of policy, management, and reporting, the lack of an accessible and broad inventory of the monetary costs of biological invasions hindered improved understanding of their burden.

Because of the lack of precise cost estimates, flawed syntheses became widely cited and led to unsubstantiated values, which, in turn, provided ammunition for denialists to challenge the legitimacy of the field. Given this background and limitations in existing cost estimates, there was a clear need for improved calibration and synthesis of the economic costs of biological invasions worldwide and, most importantly, including the traceability of sources for costs. This need resulted in the creation of an interdisciplinary team of experts from ecology, economics, and invasion sciences starting in 2014 (https://invacost.fr), who together contributed to the first public release of the *InvaCost* database (Diagne et al. 2020b).

## Advent of robust cost syntheses: Evolution of the *InvaCost* database

The *InvaCost* database is a global compilation of available estimates of the costs associated with invasive species and largely fills gaps in the standardization, description, and synthesis of reported monetary costs from biological invasions. Estimates in *InvaCost* are derived from peer-reviewed scientific articles and published grey literature, with particular attention paid to reproducibility and data descriptors. The original *InvaCost* database (v0) only included data related to the damage and management costs incurred by invasive insects, containing 260 entries (i.e., rows of data, each including a cost estimate) around the world at different spatiotemporal scales (Bradshaw et al. 2016). The first officially released version of *InvaCost* (v1.0) had grown to 2419 entries by September 2020 (doi:10.6084/m9.figshare.12668570.v1). The next publicly released version (v3.0) included 9823 entries and an associated complementary database consisting mainly of references not yet processed, as well as a database compiling data from 10 non-English language sources (Angulo et al. 2021b). In June 2021, the number of entries increased to 13,123 (v4.0), with the most recent version (v4.1, January 2022) of *InvaCost* containing 13,553 entries and compiling literature in 22 languages (Diagne et al. 2022, Kourantidou et al. 2022a). Moreover, the InvaCost project now includes a living figure that uses the latest database version to represent the most up-to-date cost breakdowns across geographic and taxonomic contexts graphically (Diagne et al. 2021a, Leroy et al. 2021).

**Figure 1.**
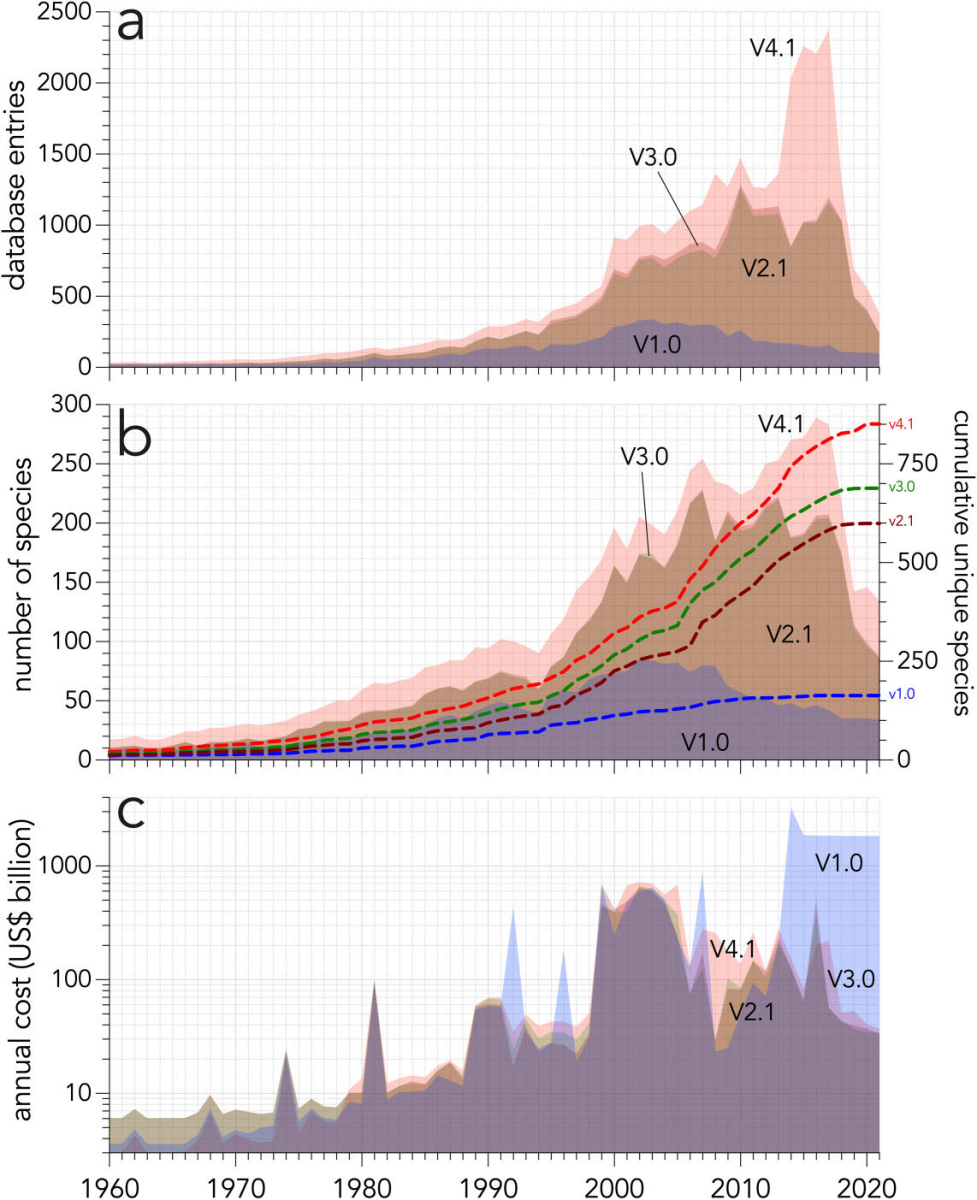
The growth of the living *InvaCost* database including entries from 1960 to 2021, showing temporal trends in (a) recorded database entries; (b) recorded species, including cumulative curves of unique species (the dashed lines, the right y axis; v1.0 is marked in blue, v2.1 in green, v3.0 in brown, and v4.1 in red; the unidentified or diverse species categories have been removed); and (c) the total costs (without any filter but expanded) among versions v1.0, v2.1, v3.0, and v4.1. The high costs associated with v1.0 in the latter years is due to refinement in subsequent versions and not filtering for cost reliability in the costs presented in the present article. The apparent logistic form of the cumulative number of unique species might arise from the reporting lag in cost data, so should not be taken to indicate saturation. R code to generate the data for these plots available at github.com/cjabradshaw/InvaCostVersionTrends. Color images are available in online versions of the article.

The ongoing growth in cost information inevitably modified summaries of cost trends reported over time (figure 1), including number of database entries (figure 1a), number of species (figure 1b), and annual costs (figure 1c). These trends each display a similar dynamic, characterized by a rapid accrual of the number of documents reporting costs, number of species reported as the cause, and the costs themselves, followed by an apparent decline in the most recent years. As with other phenomena in invasion science that have time lags (Essl et al. 2011), incurring economic costs is subject to pervasive time lags because of delays in the official reporting following species establishment and in the potential impacts after its establishment. These delays, particularly those pertaining to eventual publication of monetary cost estimates, explain the apparent decline over the most recent decades (figure 1). Several studies have accounted for these delays when summarizing temporal trends in costs (Diagne et al. 2021a, Haubrock et al. 2021) by removing or reweighting later incomplete years on the basis of the time delay between cost incurrence and reporting (Leroy et al. 2022). Nevertheless, excluding recent data or correcting for the lag in reporting as was noted by Diagne and colleagues (2021a) and Heringer and colleagues (2021), the analysis suggests that the rate and magnitude of invasion costs continue to rise.

### Capturing the multifaceted nature of invasion costs

The earlier attempts at reporting the costs of invasive species focused primarily on the United States (e.g., Pimentel et al. 2000, 2005, Pimentel 2011), whereas elsewhere, cost estimates were also provided for several other nations (including Australia, Brazil, India, South Africa, United Kingdom; Pimentel et al. 2001) and European countries (Kettunen et al. 2009). These studies were constrained not only by geography but also by taxonomic coverage, targeted sectors, and habitats.

**Figure 2.**
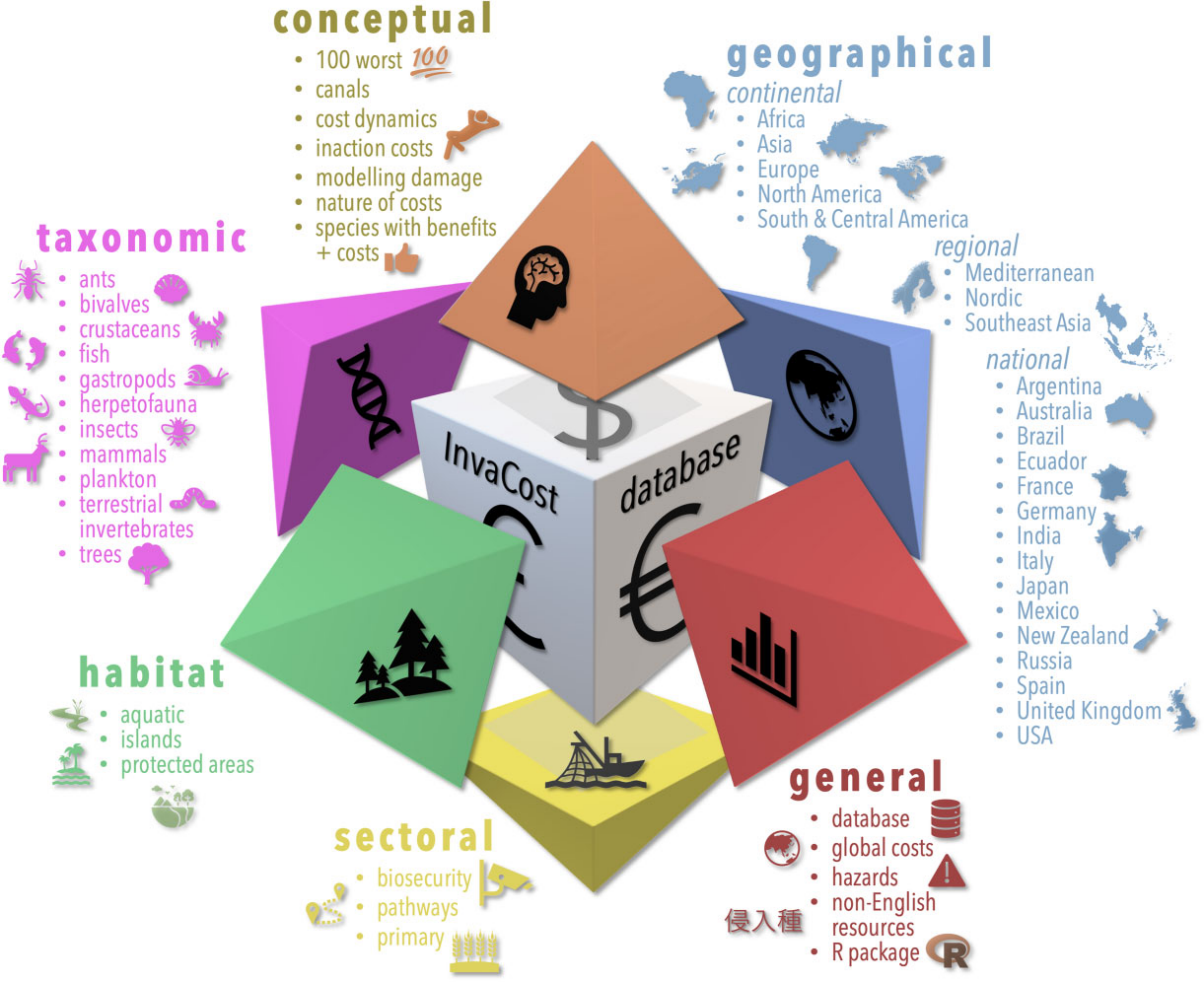
A total of 52 published research works are based on the *InvaCost* database, with studies spanning several thematic categories: taxonomic (11 studies), conceptual (7), habitat (3), sectoral (3), general (5), and geographical (23). See supplemental table S1 for references and the database version used in each study.

Recognizing and addressing such limitations, 23 publications that have used the *InvaCost* database have provided cost assessments on national, regional, or continental scales (figure 2). Of these, focus has been on nations (e.g., Spain, Angulo et al. 2021a; France, Renault et al. 2021), an important scale given it corresponds to relevant levels of governance and legal jurisdiction. Others focusing on regional scales (e.g., North America, Crystal-Ornelas et al. 2021; Europe, Haubrock et al. 2021; Mediterranean, Kourantidou et al. 2021) have provided useful comparisons between neighboring locations or trading partners that face similar invasion threats. Global-scale assessments have provided comparisons of practice across areas with differing political geographies (Bodey et al. 2022a) or common management goals (Moodley et al. 2022).

In addition, 11 studies have focused on various taxonomic groups (figure 2). Taxonomic studies can be nested at different scales, such as analyses of aquatic invaders as a whole (Cuthbert et al. 2021) followed by a detailed assessment for invasive fish (Haubrock et al. 2022a) or a compilation of costs of terrestrial invertebrates (Renault et al. 2022a) followed by a specific assessment of ants (Angulo et al. 2022). Such distinctions are useful because, although a higher level of classification can reveal the general state of knowledge, different management strategies are often needed for specific taxonomic groups, and organizations or stakeholders can have specific taxonomic foci.

For a more holistic approach, cost assessments have also been provided for other thematic categories, such as habitats (e.g., costs incurred for island conservation; Bodey et al. 2022a), sectoral (e.g., biosecurity and prevention; Cuthbert et al. 2022), conceptual (e.g., additional expenditure due to delayed action or inaction; Ahmed et al. 2022), and generic studies (e.g., increase in cost data by considering non-English sources; figure 2; Angulo et al. 2021b). Several of these InvaCost studies have solved specific problems that are applicable more broadly to scientists. For example, mathematical models illustrating the vast sums that could be saved through more proactive management investments toward biological invasions could equally be applied to other human-caused environmental changes (e.g., climate change) and emergent diseases to mitigate and reduce future problems (Ahmed et al. 2022). The value of non-English sources of data, as was illustrated by the large volume of additional data acquired in Angulo and colleagues (2021b) from 10 non-English language sources, exemplifies the substantial value in considering multilingual data sources for research syntheses. Another example is disentangling data availability from research effort using analytical approaches that measure and compare the trajectories of temporal trends, showing that biological invasion costs are increasing more rapidly than the research reporting them (Haubrock et al. 2022b).

Assessments of invasion costs at more macro scales are important because, without regional or national breakdowns of cost assessments, the data necessary to motivate national decision-making are lacking. However, these studies have revealed research gaps, particularly for cost estimation in poorly investigated regions such as in many African nations (Diagne et al. 2021b). It is also essential to contextualize and summarize costs at more granular taxonomic, sectoral, and geographically localized scales, because this information provides the detailed understanding of the impacts and costs associated with specific species or industries and can help to prioritize resources and allocate funding efficiently at subnational scales. However, such finer-scale information is often lacking at the resolutions required for effectively targeting responses (Angulo et al. 2022, Soto et al. 2022). This additional detail in reporting of costs would provide a more comprehensive understanding of the impacts of biological invasions and, ultimately, contribute to a more effective response to the threats they pose.

### Policy and communication impacts

The study on environmental and economic costs in the United States by Pimentel and colleagues (2000) has been referred to in 21 policy documents, including a report by the National Research Council (2000) and a report on evidence-based and scientifically robust risk assessments for the prevention and management of the introduction and spread of invasive species (European Commission 2022). Subsequent updates and additions have been cited in 9 (Pimentel et al. 2001) and 21 policy documents (Pimentel et al. 2005). Pimentel (2011) has not appeared in policy documents, and Kettunen and colleagues (2009) is a technical report to guide European environmental policy, with the first full assessment on different environmental, social, and economic costs and benefits across Europe.

A focus of invasion biology research is to raise awareness about the economic costs of biological invasions and to compel policymakers to increase focus on invasive species. Since its conception in 2014, scientific publications based on the *InvaCost* database have been cited in several notable policy documents. To date, 12 InvaCost studies spanning six thematic categories have been cited in at least eight policy documents or governmental reports (see supplemental table S2 for sources). A few prominent examples are general, conceptual, habitat, and geographical.

For the general category, a policy guide prepared by the Organisation for Economic Co-operation and Development on aligning budgetary and fiscal policy with biodiversity goals for G7 and other countries (Organisation for Economic Co-operation and Development 2021) cited Diagne and colleagues (2021a). The report was designed to inform finance, economic, and environment ministries, emphasizing the massive global economic costs of biological invasions, and outlining the transformative changes required to slow biodiversity loss.

For the conceptual category, a report from the European Commission that reviewed preexisting regulations on the prevention, management, and spread of invasive species highlighted the importance of timely management (European Commission 2014). The report revisited assumptions made in previous impact assessments and reaffirmed that the costs of inaction or delayed action outweigh the cost of early intervention (Ahmed et al. 2022). Also, a recent study demonstrated that the already massive economic costs of biological invasions in the European Union were considerably underestimated (Henry et al. 2023).

For the habitat category, a report by Partnerships in Environmental Management for the Seas of East Asia, which provides guidance to ministers and senior government officials from 11 partner countries (Partnerships in Environmental Management for the Seas of East Asia 2021), was focused on the regional state of oceans and coasts. That report stressed the severe economic impacts of aquatic invasive species on native fisheries, agricultural productivity, public utility operations, property values, tourism, and outdoor recreation, as well as the global expenditure of US${\$}$345 billion associated with their control since 1960 (Cuthbert et al. 2021).

For the geographical category, a scientific report compiled by the Australian Academy of Science highlighted the need for a unified national biosecurity data system and that the timely and accurate identification of invasive species is critical for environmental protection (Australian Academy of Science 2022). The report emphasized that invasive species are a serious burden to Australia with ecological, health, and economic impacts, costing an estimated AU${\$}$24.5 billion per year, citing Bradshaw and colleagues (2021).

There is an inevitable temporal lag between publication and impact of such studies through conversion into practical measures, so the aforementioned examples indicate promising progress in guiding policy and legislative decisions, and with the potential for additional policy influence to come as more studies reveal costs.

Another objective of highlighting the economic costs of invasive species is to raise public awareness tangibly about the damage caused by biological invasions (Shackleton et al. 2019). In a similar way to the costs of climate change that have been widely documented (e.g., in reports prepared by the Intergovernmental Panel on Climate Change), the costs of biological invasions are more likely to spark media interest and showcase the relevance and urgency of this issue (see supplemental table S3). Societal awareness partly determines the support, commitment, and effectiveness of public policy initiatives, because societies tend to protect only what they recognize as important (Akerlof et al. 2013). However, general awareness of biological invasions is still limited—for instance, compared with climate change (Jarić et al. 2020). As such, large-scale research into the costs of biological invasions, and most importantly, frequent scientific publications on the topic coupled with the multidisciplinarity needed to address this issue, are expected to increase its salience and relevance to media organizations, change public perception of invasive species, and improve collective support for their management (Novoa et al. 2017, Sosa et al. 2021).

In addition to their influence on policy and media uptake, cost syntheses have opened dialogues among scientists from different disciplines in academic and nonacademic organizations and institutions through both scientific publications (figure 2) and invited presentations (e.g., for NeoBiota in 2020 and for the IUCN congress in 2021). Such discussion has also enhanced method development and built an increasing consortium of researchers. Invited lectures have also been given at regional events (e.g., Alberta Invasive Species Council, Canada's Invasive Species Centre), helping disseminate results to stakeholders, citizens, and decision-makers. These events are an immediate form of engagement that can influence future long-term policy changes and research directions.

Citizen science can also be a powerful way to disseminate scientific knowledge and raise public awareness. It can be effective in achieving early detection and rapid response objectives (Howard et al. 2022), especially when it raises public awareness of problematic invasive species, and species are reported in the early stages of invasion before they can cause damage. Successful examples include the case of the network set up to monitor the invasions of two hornet species causing problems for beekeepers and wildlife: the European hornet (*Vespa crabro*) in Sardinia (Pusceddu et al. 2019) and the yellow-legged hornet (*Vespa velutina*) in Europe (Lioy et al. 2019, 2022), the latter being the object of national and international projects that include public mobilization and engagement (https://stopvelutina.it, https://vespavelutina.eu). Therefore, public engagement about the costliest species could improve their monitoring and reporting and could allow for more effective management while reducing future potential costs. Citizen-science initiatives could potentially mitigate large, negative impacts by rapidly identifying new, high-risk invaders (e.g., those on watch lists or horizon-scanning exercises) that are not yet established but that are causing damage elsewhere.

## Future directions and emerging challenges: Development of the *InvaCost* database, version 5.0

*InvaCost* is a dynamic database and has therefore evolved over time as more cost entries are added or existing ones are corrected. The living figure provides the most up-to-date, global value of the taxonomic and geographic costs of biological invasions on the basis of the latest version, v4.1 (at the time of writing, it was last updated 15 February 2022; Leroy et al. 2021). In the present article, we outline further developments that are currently in progress for the launch of *InvaCost* v5.0.

The maintenance of an up-to-date database ensures ongoing access to the most recent information, but also requires the development of assistance to users so that they can appreciate the full content and diversity of data descriptors. Improved data accessibility can assist potential users and provide a baseline for environmental decision-making. By developing dashboards where data can be visualized interactively, users can obtain their desired information quickly without requiring programming skills. The development of open-source projects such as InvaCost should ideally include tools facilitating data exploration such as shiny, an R package to build interactive web interfaces for data visualization. InvaCost members are now developing such apps that will assist in creating a more interactive environment for non-expert users.

At the same time, expertise from additional disciplines has been added to InvaCost. Previous versions of the *InvaCost* database were assembled primarily by natural scientists, but recently, social scientists (e.g., resource economists) have been working to strengthen the economic dimensions associated with the cost data collected. These new efforts encompass a more detailed analysis of the methods used for the estimation of costs in every study that will ultimately allow for an assessment of the methodology used. The need for a more granular analysis of the methods for every cost entry has arisen from the need to determine whether and to what degree the cited study is reproducible or reliable (box 1). Because the sources of costs vary (e.g., in terms of quality, estimation methods, and the nature of the costs estimated), such an analysis will provide a qualitative indicator of each of the entries in the database and will assist in specifying the evaluation methods used and the costs assessed. Beyond this, the *InvaCost* database will be developed continuously to standardize the methods of classification so that future contributors can identify these different cost characteristics for every study.

### Improving communication with managers and policymakers

The management of invasive species can often instigate social conflicts, such that managers and policymakers can be mandated to find appropriate compromises that satisfy different groups of stakeholders (Crowley et al. 2017). Conjoint considerations of costs and benefits from biological invasions have promoted dialogue to resolve conflicts among stakeholders with different economic and environmental interests (Kourantidou et al. 2022a). Moreover, the language used plays a role in effectively communicating the risks and current or potential impacts caused by invasive species to all stakeholders (Copp et al. 2021). For example, including non-English cost entries revealed communication gaps between English-speaking scientists and local practitioners (Angulo et al. 2021b). However, although information in 22 languages (including English) has been added, there remain substantial gaps, particularly across large regions of Africa and Asia, where future targeted searches would bolster outreach and the shared knowledge base. Both these language- and benefits-based initiatives could be further advanced to improve our socioeconomic understanding of costs and our ability to address how impacts affect and are distributed across stakeholders.

Future directions to improve communication among stakeholders could involve public, industry, and policy-engagement events to raise awareness of the importance and relevance of costs and open-access databases such as *InvaCost*, as well as through implementing more streamlined processes to add data from different regions, cost types, and sources. For example, the French Invasive Alien Species Resource Centre (https://especes-exotiques-envahissantes.fr) provides a dynamic spatial mapping tool of management feedback, where targeted species, the managed area and, in some instances, the cost of management and its effectiveness can be viewed. These approaches could, in turn, identify the most cost-effective species to manage, independent of their impact. A relevant analogue, *Conservation Evidence* (https://conservationevidence.com; Sutherland et al. 2019) distils scientific evidence into decision support for managers to bolster conservation by summarizing research, directing actions, and providing synopses. The expansion of such initiatives would provide a more comprehensive picture at multiple geographic scales and would facilitate the identification of knowledge gaps, thereby allowing policymakers and other stakeholders to improve and optimize strategies (e.g., ideal timing of intervention). Improved resources and better communication with decision-makers (e.g., municipalities or ministries) could provide increased awareness for better future recording or classifications of costs and more reliable cost estimates that could be incorporated in future versions of *InvaCost*. Engaging with affected industries could further catalyze actions by governments, given their operational impacts and policy influence.

Dynamic databases and the resources that flow from them allow for future corrections and updates, such as ensuring old and new taxonomies are aligned to facilitate ongoing regulation. Because invasive species require adaptive management strategies, such tools enable the assessment of the effectiveness of particular strategies across contexts (after a reasonable period that accounts for lags) and the need for refinement if conditions change (e.g., increased population sizes, ranges, or propagule pressure). This continuous calibration allows for proportionate actions while optimizing intervention and public expenditure.

### Linking *InvaCost* to other biodiversity databases

Biodiversity databases compile, collate, and standardize information on biological diversity; monitor compositional and functional changes across different levels of biological organization; provide a basis for exploring relationships between species and their environments; and identify specific geographical, taxonomic, ecological, and other knowledge gaps related to biodiversity. Although challenging, correlating data from different sources is recommended (Hobern et al. 2019, König et al. 2019). Linking the *InvaCost* database with preexisting biodiversity databases (e.g., traits, niche, and genetic data sets) has the potential to provide invasion science with better tools to quantify the links between ecological mechanisms and invasion impacts and to establish relationships among costs, ecosystems composition, and invasion dynamics (Heger et al. 2021, Ricciardi et al. 2021, Daly et al. 2023). Integrating information on costs to a broader spectrum of biological groups (not solely invasive) and invaded socioecosystems can provide insights on how invasive species affect nonnative environments, on areas in which more resources might be needed (financial, technological, or research), and on the cost efficiency of current and alternative management options. In particular, this information will enable management to be prioritized on economically vulnerable sites and pathways and on the most costly species (e.g., hypercostly species; Heringer et al. 2021) and would be effective for identifying and rapidly removing potential invasive species at the early stages of invasion (i.e., before establishment).

Over the past decade, many online databases and repositories have been established, providing digital information in thousands of primary studies on taxonomic, temporal, and spatial information, thereby facilitating broad syntheses (Hardisty et al. 2013, Guralnick et al. 2016, Dornelas et al. 2018). Although the *InvaCost* database has many similarities to such preexisting databases, *InvaCost* differs in that it serves specific purposes of standardizing costs of invasive species and therefore has a different structure, content, and functionality. One database that has been used to augment several cost analyses so far is the *Standardizing and Integrating Alien Species workflow* (Seebens et al. 2020), which includes information from five taxon-specific databases and two cross-taxon databases. The database leverages standardized biodiversity terminology (Darwin Core) to provide the most comprehensive distribution data for invasive species worldwide, with dates of first record wherever available, as well as evidence of impacts. Comparisons with *InvaCost* (Crystal-Ornelas et al. 2021) have provided estimates of missing data within the latter to test the prediction accuracy of first-record dates in invasion costs (dx.doi.org/10.21203/rs.3.rs-2444595/v1; preprint [not peer reviewed]), and measure the total management burden of invasive species (Cuthbert et al. 2022). Considering large-scale invasive species lists, such as those under the standardizing and integrating alien species workflow, there are still widespread gaps, especially in taxonomic and geographic coverage. These gaps are largely driven by discrepancies in research capacity or effort into invasive species across taxa and regions. Merging such databases with extrapolations of the real but unrecorded cost of invasions will therefore provide a more comprehensive overview of the true economic impact of invasive species.

One of the greatest challenges of connecting biodiversity databases is the lack of standardization (Feng et al. 2022), because of their particular objectives and different protocols for data acquisition and filtration, sometimes requiring specialized training (Maldonado et al. 2015). Another constraint in integrating multiple data sets is the reliability of data entries (Harris 2003) or the lack of data to sustain evidence-based actions (Dickey et al. 2020). On the other hand, monetary loss is a defined, international proxy, capable of connecting and quantifying values arising from biodiversity losses. To stimulate the next stage of data integration between *InvaCost* and multiple existing and future derivative databases, automated tools such as machine learning and artificial intelligence should be developed to integrate and analyze these different but complementary data sources (Jeschke et al. 2021, Fricke and Olden 2023). For example, machine-learning algorithms could be trained to find transcription errors in *InvaCost* from original literature sources, as well as to find additional cost sources that were not obtained in previous literature searches. Automation could also facilitate comparisons with identify taxonomic, geographic, and other research gaps across data sets, thereby providing an evidence-based tool for decision-making within and outside invasion science. A connection with the *Global Biodiversity Information Facility* (https://gbif.org) could assist with identifying cost gaps at different geographic scales and social demand by integrating citizen-science data. Merging existing databases of invasive species’ distributions with the *Global Biodiversity Information Facility* could be further leveraged for this purpose (Seebens and Kaplan 2022). For example, understanding social–ecological networks can help in the identification of opportunities for cost-effective management and the potential benefits of invasive species (Hulme et al. 2017, McGeoch and Jetz 2019).

Within and outside the context of invasive species impacts, there are many opportunities for combining this quantitative measure for social and ecological losses to produce more comprehensive databases alongside other biodiversity threats such as climate change and habitat loss or exploitation (Seebens et al. 2018, Roxburgh et al. 2020, García-León et al. 2021). These initiatives could both catalogue the direct economic impacts associated with other global changes and could be fused to examine their interactions and potential synergies (e.g., costs from biological invasions and pollution).

### Benefits alongside costs of biological invasions

Despite these recent advances in assessing the monetary costs of biological invasions at different spatial and temporal scales, the estimates are still conservative and currently capture only a fraction of total costs. These knowledge gaps occur because there are many inaccessible, nonmonetized, and missing costs, alongside costs from sources that are difficult to assess (e.g., grey literature such as internal reports; Vaissière et al. 2022), especially in non-English-speaking and low-income regions of the world. The presence of monetary benefits alongside costs has also been contested, given that *InvaCost* is only focused on assessing costs (Boltovskoy et al. 2022, Sagoff 2020, Sax et al. 2022). Part of this criticism proposes that innocuous species, ancillary costs, and failed eradication programs are potentially dominating or biassing cost analyses at present.

Currently, there is no comprehensive database that compiles the benefits of invasions as rigorously or in a way similar to what *InvaCost* does for costs. In the absence of any comparable synthesis about the benefits of biological invasions, the argument that the presence of benefits lessens the importance or risks conveyed by invasive species is therefore misleading and dangerous for conservation and human well-being. As with climate change, the establishment of biological invasions might have benefits for some. However, these benefits do not cancel out existing costs, and it is insufficient to assert the existence of these benefits without making the effort to assess them and compare them with the costs. The lack of knowledge about the magnitude, origin, and distribution of benefits of invasive species has been inappropriately used to undermine or refute existing and synthesized estimates of costs (Boltovskoy et al. 2022, Sax et al. 2022). Although it is important to quantify any simultaneous benefits alongside the costs of invasions (Kourantidou et al. 2022a), the presence of these benefits cannot be used to undermine the importance of negative impacts or costs. In fact, the presence of benefits from invasions is frequently a path-dependent outcome of the invasion itself. For example, a community might benefit today from a new species only because its introduction replaces a former native species in the region—for example, black wattle *Acacia mearnsii* (de Wit et al. 2001), Nile perch *Lates niloticus* (Aloo et al. 2017), pirarucu *Arapaima gigas* (Miranda-Chumacero et al. 2012), and other invasive fish in the Amazonia region (Doria et al. 2021). However, it is questionable whether there are any longer-term costs, particularly because of negative impacts on other species. In addition, costs and benefits are generally borne by different actors, raising problems of redistribution. Rather than claiming the existence of benefits, it is therefore essential to evaluate them to compare with the costs and, if the latter prove to be lower than the former, to ensure compensation systems. This is beyond the scope of InvaCost, whose aim is to document, collate, and synthesize knowledge about costs of biological invasions, and not to create a net cost–benefit analysis of biological invasions.

### Promoting cost reporting

InvaCost is intended to increase the quantity and quality of cost reporting. This was done initially through the catalyzing effect of high-profile studies synthesizing estimates at different biological, spatial, and sectoral scales (Zenni et al. 2021). New scientific, stakeholder, and public awareness, coupled with extensive media coverage (see table S3), encouraged further primary research to uncover additional costs, and for researchers, stakeholders, and other entities to make existing and emerging costs freely and openly available for syntheses. Templates to enter cost information into the database, alongside associated explanatory files, examples, and an email account for data submissions (updates@invacost.fr) have all improved cost reporting (Diagne et al. 2020b). This infrastructure will continue to allow new and updated cost syntheses to be constructed while raising project visibility and increasing international collaborations.

The research network has grown globally and across disciplines and has enabled many new studies synthesizing and analyzing costs. The network has grown from a core group of around a dozen scientists in 2019, to over 145 active collaborators today (https://invacost.fr/en/consortium/experts), predominantly in academia, with the network also increasing rapidly via positive feedback loops from the pools of connections harnessed from new colleagues. Although these connections have been mostly academic, there have been increasing efforts to engage with other actors (e.g., practitioners) in the gathering of (unpublished) cost data and garnering insights into the impacts of invasive species and their management. For example, although we targeted non-English materials, 1635 unpublished documents were obtained for *InvaCost* from relevant sources, such as practitioners, resource managers, and researchers (Angulo et al. 2021b). With a wealth of expertise, perspectives, and technical knowledge, these diverse colleagues renew interests in capturing costs under specific contexts related to their core research foci (taxonomic, sectoral, geographical) or employ new modelling techniques to extrapolate and new descriptors to explain documented costs. Examples include studies with new geographical focus instigated by collaborations with scientists from India (Bang et al. 2022), New Zealand (Bodey et al. 2022b), Nordic countries (Kourantidou et al. 2022b), and other assessments currently in progress (e.g., China, Turkey, South Africa). Examples that are taxon-focused include assessments of costs of invasive plankton (Macêdo et al. 2022), mammals (Wang et al. 2023), and herpetofauna (Soto et al. 2022). Aside from leading the analyses and preparing scientific articles, such projects also targeted additional collections of cost data from relevant publications, stakeholders, and organizations. They were often based on a foundation of national knowledge and existing connections, as well as breaching language and access barriers through the expertise and contacts of different collaborators.

**Figure 3.**
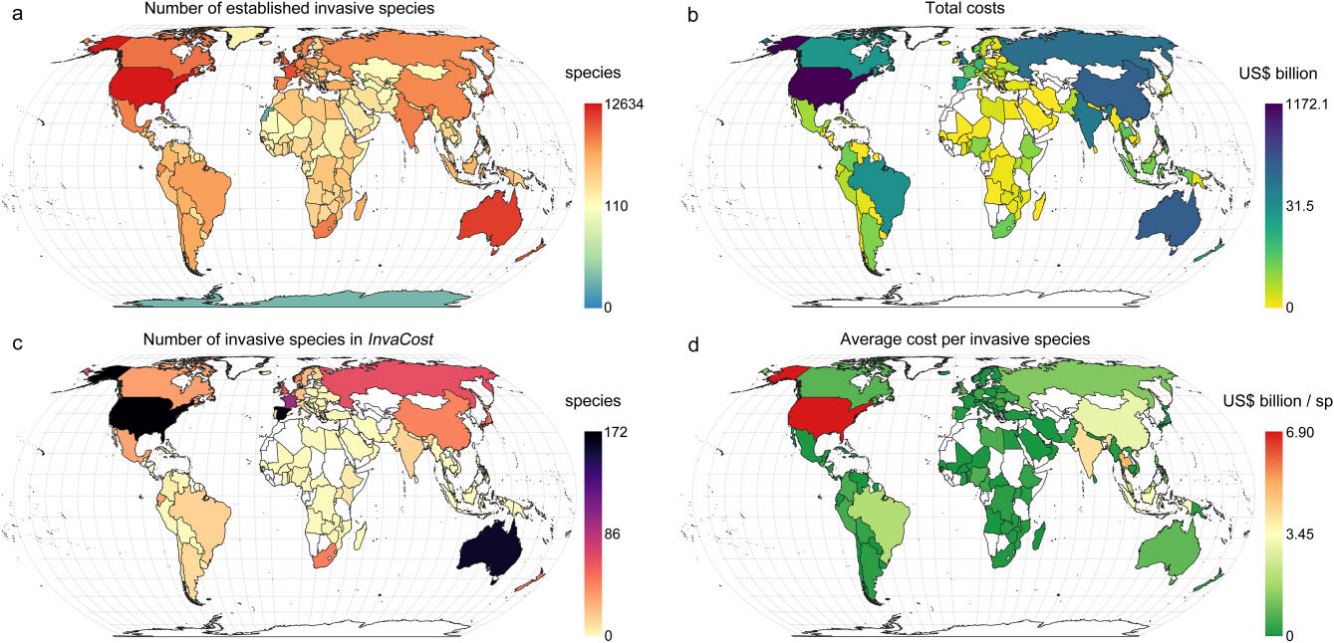
Global maps showing (a) number of established invasive species per country (see supplemental table S5; log_10_ scale), (b) total costs of invasive species (US${\$}$ billion, 2017 value; log_10_ scale) per country, (c) number of invasive species reported in *InvaCost* per country, and (d) average cost (US${\$}$ billion 2017 value) per invasive species reported in *InvaCost* in each country. The blank countries indicate an absence of data.

New research to address missing data has been promoted by InvaCost projects. Because many studies identify gaps in costs, there is now an impetus to collect these missing data. In the past, research has been hampered by a lack of syntheses, meaning that cost assessments have been partly driven by positive feedback loops—costly species are well publicized and continue to accrue scientific attention (and therefore costs; Cuthbert et al. 2021). This has contributed to poor coverage of cost assessments for most known invaders globally. Indeed, this is illustrated when comparing the total number of invasive species established per country (figure 3a) with the total costs reported in the *InvaCost* database (figure 3b), and the still low number of invasive species included therein (figure 3c). However, given the absence of costs and the disparity in values among countries (e.g., countries in Africa), the costs incurred by invasive species usually depend on the number of species with reported costs, with a few exceptions (figure 3d). A major strength in cost syntheses is therefore not only putting an aggregate monetary value on the negative impacts of biological invasions but also highlighting knowledge gaps and future research needs.

## Conclusions

The InvaCost project improves access to global and local costs arising from biological invasions, and it provides syntheses of costs across several dimensions. This information can improve targeting and prioritizing of resource investments or which species or pathways should be managed. To maximize their utility, we therefore recommend that future studies clearly specify the location, the cost per species where possible, and the period when the costs occurred. We encourage authors to adopt the nomenclature used in the *InvaCost* database (supplemental table S4; Diagne et al. 2020b), and although it is not exhaustive, it is sufficient to encompass the main aspects of the potentially affected sectors and to describe the types of cost and the management related to the time of introduction (Diagne et al. 2020b). Promoting improved granularity and quality of cost reporting has therefore been another positive outcome, helping to direct efficient allocation of resources, to foster method alignment, and to detect gaps and overlaps in cost assessments. Although InvaCost provides a solid foundation for estimating the costs of invasive species, we acknowledge that the derived estimates are merely the tip of the cost iceberg, with the biodiversity and social impacts they entail in particular requiring much additional assessment.

## Supplementary Material

biad060_Supplemental_File

## Author Biography

Danish A. Ahmed (ahmed.d@gust.edu.kw) and Phillip J. Haubrock are affiliated with the Center for Applied Mathematics and Bioinformatics, in the Department of Mathematics and Natural Sciences, at the Gulf University for Science and Technology, in Hawally, Kuwait. Phillip J. Haubrock is also affiliated with the Department of River Ecology and Conservation at the Senckenberg Research Institute and Natural History Museum Frankfurt, in Gelnhausen, Germany. Phillip J. Haubrock, Ismael Soto, Paride Balzani, and Francisco J. Oficialdegui are affiliated with the South Bohemian Research Center of Aquaculture and Biodiversity of Hydrocenoses, in the Faculty of Fisheries and Protection of Waters at the University of South Bohemia in České Budějovice, in Vodňany, in the Czech Republic. Ross N. Cuthbert is affiliated with the Institute for Global Food Security, in the School of Biological Sciences at Queen's University Belfast, in Belfast, Northern Ireland. Alok Bang is affiliated with the School of Arts and Sciences at Azim Premji University, in Bangalore, India; with the School of Arts and Sciences, Azim Premji University, in Bhopal, India; and with the Society for Ecology, Evolution, and Development, in Wardha, India. Ali Serhan Tarkan is affiliated with the Department of Basic Sciences in the Faculty of Fisheries at Muğla Sıtkı Koçman University, in Muğla, Turkey, and with the Department of Life and Environmental Sciences in the Faculty of Science and Technology at Bournemouth University, in Poole, Dorset, England, in the United Kingdom. Rafael L. Macêdo is affiliated with the Graduate Program in Conservation and Ecotourism at the Federal University of Rio de Janeiro State, in Rio de Janeiro, Rio de Janeiro State, Brazil; with the Institute of Biology at Freie Universität Berlin, in Berlin, Germany; and with the Neotropical Limnology Group, at the Federal University of Rio de Janeiro State, in Rio de Janeiro, Rio de Janeiro State, Brasil. Laís Carneiro is affiliated with the Laboratório de Ecologia e Conservação in the Departamento de Engenharia Ambiental, Setor de Tecnologia, at the Universidade Federal do Paraná, in Curitiba, Paraná, Brazil. Thomas W. Bodey is affiliated with the School of Biological Sciences at King's College, at the University of Aberdeen, in Aberdeen, Scotland, in the United Kingdom. Pierre Courtois is affiliated with the Centre for Environmental Economics—Montpellier, at the National Institute for Research in Agriculture and the Environment, in Montpellier, France. Melina Kourantidou is affiliated with the Department of Sociology, Environmental and Business Economics at the University of Southern Denmark, in Esbjerg Ø, Denmark, and with the Université de Bretagne Occidentale, in Plouzané, France. Elena Angulo is affiliated with the Estación Biológica de Doñana, in Seville, Spain. Gustavo Heringer is affiliated with the Departamento de Ecologia e Conservação in the Instituto de Ciências Naturais at the Universidade Federal de Lavras, in Lavras, Minas Gerais, in Brazil, and with Nürtingen-Geislingen University, in Nürtingen, Germany. David Renault is affiliated with the Centre National de Recherche Scientifique's Ecosystèmes, Biodiversité, Evolution, at the University of Rennes, in Rennes, France. Anna J. Turbelin, Ugo Arbieu, and Franck Courchamp are affiliated with the Université Paris–Saclay, CNRS, AgroParisTech, Ecologie Systématique Evolution, in Gif-sur-Yvette, France. Anna J. Turbelin is also affiliated with the Great Lakes Forestry Centre at Canadian Forestry Services, part of Natural Resources Canada, in Sault Ste Marie, Ontario, Canada. Emma J. Hudgins is affiliated with the Department of Biology at Carleton University, in Ottawa, Ontario, Canada. Chunlong Liu is affiliated with the College of Fisheries at the Ocean University of China, in Qingdao, and with the Institute of Hydrobiology at the Chinese Academy of Sciences, in Wuhan, China. Showkat A. Gojery is affiliated with the Department of Botany at the University of Kashmir, in Kashmir, India. Ugo Arbieu is also affiliated with the Senckenberg Biodiversity and Climate Research Centre, in Frankfurt am Main, Germany, and with the Smithsonian Conservation Biology Institute, at the National Zoological Park, in Front Royal, Virginia, in the United States. Christophe Diagne is affiliated with the Centre de Biologie pour la Gestion des Populations, at Institut de Recherche pour le Développement, in Montferrier-sur-Lez Cedex, France. Boris Leroy is affiliated with the Unité Biologie des Organismes et des Ecosystèmes Aquatiques, at the Muséum National d’Histoire Naturelle, Sorbonne Universités, Université de Caen Normandie, Université des Antilles, in Paris, France. Elizabeta Briski is affiliated with the GEOMAR Helmholtz-Zentrum für Ozeanforschung Kiel, in Kiel, Germany. Corey J. A. Bradshaw is affiliated with the Global Ecology Laboratory, *Partuyarta Ngadluku Wardli Kuu*, at the College of Science and Engineering, at Flinders University, in Adelaide, South Australia, and with the ARC Centre of Excellence for Australian Biodiversity and Heritage, in Wollongong, New South Wales, Australia.
