# Supplementary material for: Recent advances in availability and synthesis of the economic costs of biological invasions
Source: Bioscience. 2023 Aug 22;73(8):560–74. doi: 10.1093/biosci/biad060 (PMC10481418; doi:10.1093/biosci/biad060)
Supplement: biad060_Supplemental_File [file biad060_supplemental_file.docx]

| **Supporting Information for**  **Recent advances in availability and synthesis of the economic costs of biological invasions** | | | | | | | | | | |  |  |  |
| --- | --- | --- | --- | --- | --- | --- | --- | --- | --- | --- | --- | --- | --- |
|  |  |  |  |  |  |  |  |  | |  | |  |  |
| Ahmed DA, Haubrock PJ, Cuthbert RN, Bang A, Soto I, Balzani P, Tarkan AS, Macêdo RL, Carneiro L, Bodey TW, Oficialdegui FJ, Courtois P, Kourantidou M, Angulo E, Heringer G, Renault D, Turbelin AJ, Hudgins EJ, Liu C, Gojery SA, Arbieu U, Diagne C, Leroy B, Briski E, Bradshaw CJA, Courchamp F | | | | | | | | |  | | | | |
| Tables S1–S4 also available as .csv files at github.com/cjabradshaw/InvaCostVersionTrends | | | | | | | | |  | | | | |
|  |  |  |  |  |  |  |  |  | |  | |  |  |
| Updated on 9^th^ March 2023 | | |  |  |  |  |  |  | |  | |  |  |

| **Table S1 InvaCost publications** List of 52 peer-reviewed articles that have used the InvaCost database, categorised according to themes: taxonomic (11), conceptual (7), habitat (3), sectoral (3) and geographical (23; regional, continental and national). The studies, trees (Fernandez et al. 2022), primary (Turbelin et al. 2023b) are available in preprint repositories, and hazards (Turbelin et al. 2023a) is 'in press'. All other studies are published in journals. | | | | |  |
| --- | --- | --- | --- | --- | --- |
|  |  |  |  |  |  |
| **taxonomic** | | | | |  |
| **no.** | **category** | **citation** | **reference** | **version** |  |
| 1 | ants | Angulo et al. (2022) | Angulo, E., Hoffmann, B.D., Ballesteros-Mejia, L., Taheri, A., Balzani, P., Bang, A., et al. (2022). Economic costs of invasive alien ants worldwide. Biol. Invasions, 24, 2041–2060 doi:10.1007/s10530-022-02791-w. | 4 |  |
| 2 | bivalves | Haubrock et al. (2022c) | Haubrock, P.J., Cuthbert, R.N., Ricciardi, A., Diagne, C. & Courchamp, F. (2022). Economic costs of invasive bivalves in freshwater ecosystems. Divers. Distrib., 28, 1010–1021 doi:10.1111/ddi.13501 | 4 |  |
| 3 | crustaceans | Kouba et al. (2022) | Kouba, A., Oficialdegui, F.J., Cuthbert, R.N., Kourantidou, M., South, J., Tricarico, E., et al. (2022). Identifying economic costs and knowledge gaps of invasive aquatic crustaceans. Sci. Total Environ., 813, 152325 doi:10.1016/j.scitotenv.2021.152325 | 4 |  |
| 4 | fish | Haubrock et al. (2022a) | Haubrock, P.J., Bernery, C., Cuthbert, R.N., Liu, C., Kourantidou, M., Leroy, B., et al. (2022). Knowledge gaps in economic costs of invasive alien fish worldwide. Sci. Total Environ., 803, 149875 doi:10.1016/j.scitotenv.2021.149875 | 4 |  |
| 5 | gastropods | Jiang et al. (2022) | Jiang, X., Zheng, P., Soto, I., Haubrock, P.J., Chen, J. & Ji, L. (2022). Global economic costs and knowledge gaps of invasive gastropods. Ecol. Indic., 145, 109614 doi:10.1016/j.ecolind.2022.109614 | 4.1 |  |
| 6 | herpetofauna | Soto et al. (2022) | Soto, I., Cuthbert, R.N., Kouba, A., Capinha, C., Turbelin, A., Hudgins, E.J., et al. (2022). Global economic costs of herpetofauna invasions. Sci. Rep., 12, 10829 doi:10.1038/s41598-022-15079-9 | 4 |  |
| 7 | insects | Bradshaw et al. (2016) | Bradshaw, C.J.A., Leroy, B., Bellard, C., Roiz, D., Albert, C., Fournier, A., et al. (2016). Massive yet grossly underestimated global costs of invasive insects. Nat. Commun., 7, 12986 doi:10.1038/ncomms12986 | 0 |  |
| 8 | mammals | Wang et al. (2023) | Wang, S., Deng, T., Zhang, J. & Li, Y. (2023). Global economic costs of mammal invasions. Sci. Total Environ., 857, 159479 doi:10.1016/j.scitotenv.2022.159479 | 4.1 |  |
| 9 | plankton | Macêdo et al. (2022) | Macêdo, R.L., Franco, A.C.S., Kozlowsky-Suzuki, B., Mammola, S., Dalu, T. & Rocha, O. (2022). The global social-economic dimension of biological invasions by plankton: grossly underestimated costs but a rising concern for water quality benefits? Water Res., 222, 118918 doi:10.1016/j.watres.2022.118918 | 4 |  |
| 10 | trees | Fernandez et al. (2022) | Fernandez, R.D., Haubrock, P.J., Cuthbert, R., Heringer, G., Kourantidou, M., Hudgins, E.J., et al. (2022). Underexplored and growing economic costs of invasive alien trees. SSRN doi:10.2139/ssrn.4196468 | 4.1 |  |
| 11 | terrestrial invertebrates | Renault et al. (2022) | Renault, D., Angulo, E., Cuthbert, R.N., Haubrock, P.J., Capinha, C., Bang, A., et al. (2022). The magnitude, diversity, and distribution of the economic costs of invasive terrestrial invertebrates worldwide. Sci. Total Environ., 835, 155391 doi:10.1016/j.scitotenv.2022.155391 | 4.1 |  |
|  |  |  |  |  |  |
| **conceptual** | | | | |  |
| **no.** | **category** | **citation** | **reference** | **version** |  |
| 1 | 100 worst | Cuthbert et al. (2022a) | Cuthbert, R.N., Diagne, C., Haubrock, P.J., Turbelin, A.J. & Courchamp, F. (2022). Are the “100 of the world’s worst” invasive species also the costliest? Biol. Invasions, 24, 1895–1904 doi:10.1007/s10530-021-02568-7 | 3 |  |
| 2 | canals | Balzani et al. (2022) | Balzani, P., Cuthbert, R.N., Briski, E., Galil, B., Castellanos-Galindo, G.A., Kouba, A., et al. (2022). Knowledge needs in economic costs of invasive species facilitated by canalisation. NeoBiota, 78, 207–223 doi:10.3897/neobiota.78.95050 | 4.1 |  |
| 3 | cost dynamics | Haubrock et al. (2022b) | Haubrock, P.J., Cuthbert, R.N., Hudgins, E.J., Crystal-Ornelas, R., Kourantidou, M., Moodley, D., et al. (2022). Geographic and taxonomic trends of rising biological invasion costs. Sci. Total Environ., 817, 152948 doi:10.1016/j.scitotenv.2022.152948 | 3 |  |
| 4 | inaction costs | Ahmed et al. (2022b) | Ahmed, D.A., Hudgins, E.J., Cuthbert, R.N., Kourantidou, M., Diagne, C., Haubrock, P.J., et al. (2022). Managing biological invasions: the cost of inaction. Biol. Invasions, 24, 1927–1946 doi:10.1007/s10530-022-02755-0 | 4 |  |
| 5 | modelling damage costs | Ahmed et al. (2022a) | Ahmed, D.A., Hudgins, E.J., Cuthbert, R.N., Haubrock, P.J., Renault, D., Bonnaud, E., et al. (2022). Modelling the damage costs of invasive alien species. Biol. Invasions, 24, 1949–1972 doi:10.1007/s10530-021-02586-5 | 1 |  |
| 6 | nature of costs | Vaissière et al. (2022) | Vaissière, A.-C., Courtois, P., Courchamp, F., Kourantidou, M., Diagne, C., Essl, F., et al. (2022). The nature of economic costs of biological invasions. Biol. Invasions, 24, 2081–2101 doi:10.1007/s10530-022-02837-z | 4 |  |
| 7 | species with benefits + costs | Kourantidou et al. (2022a) | Kourantidou, M., Haubrock, P.J., Cuthbert, R.N., Bodey, T.W., Lenzner, B., Gozlan, R.E., et al. (2022). Invasive alien species as simultaneous benefits and burdens: trends, stakeholder perceptions and management. Biol. Invasions, 24, 1905–1926 doi:10.1007/s10530-021-02727-w | 4 |  |
|  |  |  |  |  |  |
| **habitat** | | | | |  |
| **no.** | **category** | **citation** | **reference** | **version** |  |
| 1 | aquatic | Cuthbert et al. (2021b) | Cuthbert, R.N., Pattison, Z., Taylor, N.G., Verbrugge, L., Diagne, C., Ahmed, D.A., et al. (2021). Global economic costs of aquatic invasive alien species. Sci. Total Environ., 775, 145238 doi:10.1016/j.scitotenv.2021.145238 | 3 |  |
| 2 | islands | Bodey et al. (2022a) | Bodey, T.W., Angulo, E., Bang, A., Bellard, C., Fantle-Lepczyk, J., Lenzner, B., et al. (2022). Economic costs of protecting islands from invasive alien species. Conserv. Biol., e14034 doi:10.1111/cobi.14034 | 4 |  |
| 3 | protected areas | Moodley et al. (2022) | Moodley, D., Angulo, E., Cuthbert, R.N., Leung, B., Turbelin, A., Novoa, A., et al. (2022). Surprisingly high economic costs of biological invasions in protected areas. Biol. Invasions, 24, 1995–2016 doi:10.1007/s10530-022-02732-7 | 4 |  |
|  |  |  |  |  |  |
| **sectoral** | | | | |  |
| **no.** | **category** | **citation** | **reference** | **version** |  |
| 1 | biosecurity | Cuthbert et al. (2022b) | Cuthbert, R.N., Diagne, C., Hudgins, E.J., Turbelin, A., Ahmed, D.A., Albert, C., et al. (2022). Biological invasion costs reveal insufficient proactive management worldwide. Sci. Total Environ., 819, 153404 doi:10.1016/j.scitotenv.2022.153404 | 4 |  |
| 2 | pathways | Turbelin et al. (2022) | Turbelin, A.J., Diagne, C., Hudgins, E.J., Moodley, D., Kourantidou, M., Novoa, A., et al. (2022). Introduction pathways of economically costly invasive alien species. Biol. Invasions, 24, 2061–2079 doi:10.1007/s10530-022-02796-5 | 4 |  |
| 3 | primary | Turbelin et al. (2023b) | Turbelin, A.J., Hudgins, E.J., Catford, J.A., Cuthbert, R.N., Kourantidou, M., Roiz, D., et al. (2023). Biological invasions as burdens to primary economic sectors. Res. Sq. doi:10.21203/rs.3.rs-2444595/v1 | 4.1 |  |
|  |  |  |  |  |  |
| **general** | | | | |  |
| **no.** | **category** | **citation** | **reference** | **version** |  |
| 1 | database | Diagne et al. (2020) | Diagne, C., Leroy, B., Gozlan, R.E., Vaissière, A.-C., Assailly, C., Nuninger, L., et al. (2020). InvaCost, a public database of the economic costs of biological invasions worldwide. Sci. Data, 7, 277 doi:10.1038/s41597-020-00586-z | 1 |  |
| 2 | global costs | Diagne et al. (2021a) | Diagne, C., Leroy, B., Vaissière, A.-C., E. Gozlan, R., Roiz, D., Jarić, I., et al. (2021). High and rising economic costs of biological invasions worldwide. Nature, 592, 571–576 doi:10.1038/s41586-021-03405-6 | 1 |  |
| 3 | hazards | Turbelin et al. (2023a) | Turbelin, A.J., Cuthbert, R.N., Essl, F., Haubrock, P.J., Ricciardi, A. & Courchamp, F. (2023a). Biological invasions are as costly as natural hazards. Perspect. Ecol. Conserv. doi: 10.1016/j.pecon.2023.03.002 | 4.1 |  |
| 4 | non-English sources | Angulo et al. (2021b) | Angulo, E., Diagne, C., Ballesteros-Mejia, L., Adamjy, T., Ahmed, D.A., Akulov, E., et al. (2021). Non-English languages enrich scientific knowledge: the example of economic costs of biological invasions. Sci. Total Environ., 775, 144441 doi: 10.1016/j.scitotenv.2020.144441 | 3 |  |
| 5 | R package | Leroy et al. (2022) | Leroy, B., Kramer, A.M., Vaissière, A.-C., Kourantidou, M., Courchamp, F. & Diagne, C. (2022). Analysing economic costs of invasive alien species with the invacost R package. Methods Ecol. Evol., 13, 1930–1937 doi:10.1111/2041-210X.13929 | 4 |  |
|  |  |  |  |  |  |
| **geographical** | | | | |  |
| **no.** | **category** | **citation** | **reference** | **version** |  |
| ***continental*** | | | | |  |
| 1 | Africa | Diagne et al. (2021b) | Diagne, C., Turbelin, A.J., Moodley, D., Novoa, A., Leroy, B., Angulo, E., et al. (2021). The economic costs of biological invasions in Africa: a growing but neglected threat? NeoBiota, 67, 11–51 doi:10.3897/neobiota.67.59132 | 3 |  |
| 2 | Asia | Liu et al. (2021) | Liu, C., Diagne, C., Angulo, E., Banerjee, A.-K., Chen, Y., Cuthbert, R.N., et al. (2021). Economic costs of biological invasions in Asia. NeoBiota, 67, 53–78 doi:10.3897/neobiota.67.58147 | 1 |  |
| 3 | Europe | Haubrock et al. (2021d) | Haubrock, P.J., Turbelin, A.J., Cuthbert, R.N., Novoa, A., Taylor, N.G., Angulo, E., et al. (2021). Economic costs of invasive alien species across Europe. NeoBiota, 67, 153–190 doi:10.3897/neobiota.67.58196 | 1 |  |
| 4 | North America | Crystal-Ornelas et al. (2021) | Crystal-Ornelas, R., Hudgins, E.J., Cuthbert, R.N., Haubrock, P.J., Fantle-Lepczyk, J., Angulo, E., et al. (2021). Economic costs of biological invasions within North America. NeoBiota, 67, 485–510 doi:10.3897/neobiota.67.58038 | 3 |  |
| 5 | South and Central America | Heringer et al. (2021) | Heringer, G., Angulo, E., Ballesteros-Mejia, L., Capinha, C., Courchamp, F., Diagne, C., et al. (2021). The economic costs of biological invasions in Central and South America: a first regional assessment. NeoBiota, 67, 401–426 doi:10.3897/neobiota.67.59193 | 3 |  |
| ***regional*** | | | | |  |
| 6 | Mediterranean | Kourantidou et al. (2021) | Kourantidou, M., Cuthbert, R.N., Haubrock, P.J., Novoa, A., Taylor, N.G., Leroy, B., et al. (2021). Economic costs of invasive alien species in the Mediterranean basin. NeoBiota, 67, 427–458 doi:10.3897/neobiota.67.58926 | 3 |  |
| 7 | Nordic | Kourantidou et al. (2022b) | Kourantidou, M., Verbrugge, L.N.H., Haubrock, P.J., Cuthbert, R.N., Angulo, E., Ahonen, I., et al. (2022). The economic costs, management and regulation of biological invasions in the Nordic countries. J. Environ. Manage., 324, 116374 doi:10.1016/j.jenvman.2022.116374 | 4.1 |  |
| 8 | Southeast Asia | Haubrock et al. (2021c) | Haubrock, P.J., Cuthbert, R.N., Yeo, D.C.J., Banerjee, A.K., Liu, C., Diagne, C., et al. (2021). Biological invasions in Singapore and Southeast Asia: data gaps fail to mask potentially massive economic costs. NeoBiota, 67, 131–152 doi:10.3897/neobiota.67.64560 | 3 |  |
| ***national*** | | | | |  |
| 9 | Argentina | Duboscq-Carra et al. (2021) | Duboscq-Carra, V.G., Fernandez, R.D., Haubrock, P.J., Dimarco, R.D., Angulo, E., Ballesteros-Mejia, L., et al. (2021). Economic impact of invasive alien species in Argentina: a first national synthesis. NeoBiota, 67, 329–348 doi:10.3897/neobiota.67.63208 | 3 |  |
| 10 | Australia | Bradshaw et al. (2021a) | Bradshaw, C.J.A., Hoskins, A.J., Haubrock, P.J., Cuthbert, R.N., Diagne, C., Leroy, B., et al. (2021). Detailed assessment of the reported economic costs of invasive species in Australia. NeoBiota, 67, 511–550 doi:10.3897/neobiota.67.58834 | 1 |  |
| 11 | Brazil | Adelino et al. (2021) | Adelino, J.R.P., Heringer, G., Diagne, C., Courchamp, F., Faria, L.D.B. & Zenni, R.D. (2021). The economic costs of biological invasions in Brazil: a first assessment. NeoBiota, 67, 349–374 doi:10.3897/neobiota.67.59185 | 3 |  |
| 12 | Ecuador | Ballesteros-Mejia et al. (2021) | Ballesteros-Mejia, L., Angulo, E., Diagne, C., Cooke, B., Nuñez, M.A. & Courchamp, F. (2021). Economic costs of biological invasions in Ecuador: the importance of the Galapagos Islands. NeoBiota, 67, 375–400 doi:10.3897/neobiota.67.59116 | 3 |  |
| 13 | France | Renault et al. (2021) | Renault, D., Manfrini, E., Leroy, B., Diagne, C., Ballesteros-Mejia, L., Angulo, E., et al. (2021). Biological invasions in France: alarming costs and even more alarming knowledge gaps. NeoBiota, 67, 191–224 doi:10.3897/neobiota.67.59134 | 3 |  |
|  |  |  |  |  |  |
| 14 | Germany | Haubrock et al. (2021a) | Haubrock, P.J., Cuthbert, R.N., Sundermann, A., Diagne, C., Golivets, M. & Courchamp, F. (2021). Economic costs of invasive species in Germany. NeoBiota, 67, 225–246 doi:10.3897/neobiota.67.59502 | 3 |  |
| 15 | India | Bang et al. (2022) | Bang, A., Cuthbert, R.N., Haubrock, P.J., Fernandez, R.D., Moodley, D., Diagne, C., et al. (2022). Massive economic costs of biological invasions despite widespread knowledge gaps: a dual setback for India. Biol. Invasions, 24, 2017–2039 doi:10.1007/s10530-022-02780-z | 3 |  |
| 16 | Italy | Haubrock et al. (2021b) | Haubrock, P.J., Cuthbert, R.N., Tricarico, E., Diagne, C., Courchamp, F. & Gozlan, R.E. (2021). The recorded economic costs of alien invasive species in Italy. NeoBiota, 67, 247–266 doi:10.3897/neobiota.67.57747 | 3 |  |
| 17 | Japan | Watari et al. (2021) | Watari, Y., Komine, H., Angulo, E., Diagne, C., Ballesteros-Mejia, L. & Courchamp, F. (2021). First synthesis of the economic costs of biological invasions in Japan. NeoBiota, 67, 79–101 doi:10.3897/neobiota.67.59186 | 3 |  |
| 18 | Mexico | Rico-Sánchez et al. (2021) | Rico-Sánchez, A.E., Haubrock, P.J., Cuthbert, R.N., Angulo, E., Ballesteros-Mejia, L., López-López, E., et al. (2021). Economic costs of invasive alien species in Mexico. NeoBiota, 67, 459–483 doi:10.3897/neobiota.67.63846 | 3 |  |
| 19 | New Zealand | Bodey et al. (2022b) | Bodey, T.W., Carter, Z.T., Haubrock, P.J., Cuthbert, R.N., Welsh, M.J., Diagne, C., et al. (2022). Building a synthesis of economic costs of biological invasions in New Zealand. PeerJ, 10, e13580 doi:10.7717/peerj.13580 | 4 |  |
| 20 | Russia | Kirichenko et al. (2021) | Kirichenko, N., Haubrock, P.J., Cuthbert, R.N., Akulov, E., Karimova, E., Shneider, Y., et al. (2021). Economic costs of biological invasions in terrestrial ecosystems in Russia. NeoBiota, 67, 103–130 doi:10.3897/neobiota.67.58529 | 1 |  |
| 21 | Spain | Angulo et al. (2021a) | Angulo, E., Ballesteros-Mejia, L., Novoa, A., Duboscq-Carra, V.G., Diagne, C. & Courchamp, F. (2021a). Economic costs of invasive alien species in Spain. NeoBiota, 67, 267–297 doi:10.3897/neobiota.67.59181 | 3 |  |
| 22 | United Kingdom | Cuthbert et al. (2021a) | Cuthbert, R.N., Bartlett, A.C., Turbelin, A.J., Haubrock, P.J., Diagne, C., Pattison, Z., et al. (2021). Economic costs of biological invasions in the United Kingdom. NeoBiota, 67, 299–328 doi:10.3897/neobiota.67.59743 | 3 |  |
| 23 | USA | Fantle-Lepczyk et al. (2022) | Fantle-Lepczyk, J.E., Haubrock, P.J., Kramer, A.M., Cuthbert, R.N., Turbelin, A.J., Crystal-Ornelas, R., et al. (2022). Economic costs of biological invasions in the United States. Sci. Total Environ., 806, 151318 doi:10.1016/j.scitotenv.2021.151318 | 3 |  |

| **Table S2** Compilation of policy documents that have referenced InvaCost studies. See also supporting information Table S1 for complete citation for each InvaCost study. | | | | | | | | | |  |  |  |
| --- | --- | --- | --- | --- | --- | --- | --- | --- | --- | --- | --- | --- |
|  | |  | |  | | |  | |  |  |  |  |
| **policy document (citation)** | | **published date** | | **link (DOI/URL)** | | | **InvaCost studies cited (citation)** | | **thematic category** |  |  |  |
| OECD (2021), "Biodiversity, natural capital and the economy: A policy guide for finance, economic and environment ministers", OECD Environment Policy Papers, No. 26, OECD Publishing, Paris. | | May-21 | | doi:10.1787/1a1ae114-en | | | Diagne et al. (2021a), global | | general |  |  |  |
| Australian Academy of Science (2022). Australia’s data-enabled research future: Science. | | Jun-22 | | science.org.au/supporting-science/science-policy-and-analysis/reports-and-publications/australias-data-enabled-research-future-science | | | Bradshaw et al. (2021a), Australia | | geographical |  |  |  |
| European Commission, Directorate-General for Environment, Nesbit, M., Whiteoak, K., Underwood, E., et al., Biodiversity financing and tracking : final report, Publications Office of the European Union, 2022. | | May-22 | | doi:10.2779/950856 | | | Haubrock et al. (2021d), Europe | | geographical |  |  |  |
| IPPC Secretariat. 2022. Report on the analysis to support the transition to a sustainable Implementation Review and Support System (IRSS). Rome, FAO on behalf of the Secretariat of the International Plant Protection Convention. | | Sep-22 | | doi:10.4060/cc0799en | | | Cuthbert et al. (2021b), aquatic | | habitat |  |  |  |
| Report from the commission to the EU parliament and the council on the review of the application of Regulation (EU) No 1143/2014 of the European Parliament and of the Council of 22 October 2014 on the prevention and management of the introduction and spread of invasive alien species. | | Oct-21 | | eur-lex.europa.eu/legal-content/EN/TXT/?uri=CELEX:52021DC0628 | | | Diagne et al. (2021a), global | | general |  |  |  |
|  |  |  |  |  |  |  | Cuthbert et al. (2021b), aquatic | | habitat |  |  |  |
|  |  |  |  |  |  |  | Ahmed et al. (2022b), inaction costs | | conceptual |  |  |  |
| Regional State of Ocean and Coasts 2021: The East Asian Seas Region (Volume 1). | | Jun-22 | | app.overton.io/document.php?policy_document_id=pemsea-30680e5825f82040e533f9a66eff2dd9 | | | Cuthbert et al. (2021b), aquatic | | habitat |  |  |  |
| Manual of invasive alien species in the Eastern Mediterranean. IUCN Centre for Mediterranean Cooperation. IUCN, Regional Office for West Asia (ROWA) | | 2023 | | https://www.iucn.org/resources/grey-literature/manual-invasive-alien-species-eastern-mediterranean | | | Cuthbert et al. (2021b), aquatic  Cuthbert et al. (2022b), biosecurity | | habitat  sectoral |  |  |  |
| European Commission, Directorate-General for Environment, Study on invasive alien species : development of risk assessments to tackle priority species and enhance prevention : final report, Publications Office of the European Union, 2022. | | Dec-22 | | https://data.europa.eu/doi/10.2779/5726 | | | Kouba et al. (2022), crustaceans | | taxonomic |  |  |  |
|  |  |  |  |  |  |  | Angulo et al. (2022), ants | | taxonomic |  |  |  |
| **Table S3 News articles** Altmetric scores, examples of news stories with associated outlets, and number of tweets. The examples provided are those captured either by Altmetric or PlumX and therefore the media coverage listed below is not exhaustive.  The Altmetric tool is a useful tool to measure the media ‘attention’ received by scholarly articles, based on the volume, importance of the sources, and the authors of media publications, and blog and social network posts (see Supplementary Information Table S3 for details on each InvaCost-related study). These descriptive statistics highlight the breadth of policy reports, media outlets, and social-network discussions that InvaCost studies have generated online, demonstrating a current momentum for putting biological invasions on the media agenda. Some studies have been prominently featured in the media landscape: 8 studies received an Altmetric score > 100, which, when put in the context of the Altmetric performance of their respective journals, correspond to the top 2% of scientific publications tracked by Altmetric (Angulo et al. 2022, Bradshaw et al. 2021a, Bradshaw et al. 2016, Cuthbert et al. 2022a, Cuthbert et al. 2021b, Diagne et al. 2020, Diagne et al. 2021a, Soto et al. 2022). From the earlier works on economic costs of invasive species, the study by Pimentel et al. (2000) has an Altmetric score of < 100, but this value is not comparable because this can only be done for research outputs of a similar age or source (Elmore 2018).  Media outreach has also been achieved through the production of magazine articles aimed at scientists, politicians, and the public. Examples include an article on the economic costs of aquatic invasions in *Eco Magazine* (Eco 2021) and *World Fishing and Aquaculture* (World Fishing and Aquaculture 2021), features in *Science* *for Environment Policy* (European Commission Directorate-General for Environment 2022) to inform management strategies, as well as *The Environment* (Cuthbert 2022)and *The Conversation* (Bernery et al. 2021, Bradshaw and Hoskins 2021, Bradshaw et al. 2021b, Courchamp 2021, Soto and Hudgins 2022). Such evidence underscores the importance, interest, and applicability of assessing the costs of invasive species for end users. | | | | | | | | | | | | |
|  |  | |  | |  |  | |  | | |  |  |
| **taxonomic** | | | | | | | | | | | | |
| **no.** | **category** | | **citation** | | **Altmetric (or PlumX)** | **news story examples** | | **associated outlet(s)** | | | **news stories (outlets)** | **tweets (users)** |
| 1 | ants | | Angulo et al. (2022) | | 101 | Le formiche invasive sono una minaccia più grande del previsto | | *National Geographic* | | | 6 (5) | 96 (86) |
|  |  |  |  |  |  | Invasive ants are a bigger threat than we thought | | *NewsBeezer*, *MSN*, *National Geographic* | | |  |  |
|  |  |  |  |  |  | Cuantifican los efectos de las hormigas invasoras | | *Catalunya Avanguardista* | | |  |  |
|  |  |  |  |  |  | El coste económico provocado por el impacto de las hormigas invasoras alcanza los 46.000 millones de euros | | *Dicyt* | | |  |  |
| 2 | crustaceans | | Kouba et al. (2022) | | PlumX | The Signal Crayfish: The Cost of Invasion | | *The Orkney News* | | | 2 (2) | - |
|  |  |  |  |  |  | Hohe wirtschaftliche Schäden durch invasive Krebstiere | | *Wissenschaft.de* | | |  |  |
| 3 | fish | | Haubrock et al. (2022a) | | PlumX | - | | - | | | - | 28 |
| 4 | gastropods | | Jiang et al. (2022) | | PlumX | - | | - | | | - | 16 |
| 5 | herpetofauna | | Soto et al. (2022) | | 626 | BEYOND LOCAL: Invasive species are causing billions of dollars in damages globally | | *TimminsToday.com* | | | 98 (76) | 40 (35) |
|  |  |  |  |  |  | Costs of amphibian and reptile invasions exceeded US$17 billion between 1986 and 2020 | | *Phys.org, Swift Telecast, Nature Asia, WhatsNew, Environewsbits* | | |  |  |
|  |  |  |  |  |  | Environment: Costs of amphibian and reptile invasions exceeded US$ 17 billion between 1986 and 2020 | | *EurekAlert!* | | |  |  |
|  |  |  |  |  |  | Invasive reptiles have cost the global economy billions | | *Earth* | | |  |  |
|  |  |  |  |  |  | Invasive species bullfrog and snake cost world $16bn - study | | *BBC News* | | |  |  |
| 6 | insects | | Bradshaw et al. (2016) | | 622 | Invasive insects cost the world billions per year | | *Seed Daily, Environment Institute blog* | | | 70 (57) | 247 (209) |
|  |  |  |  |  |  | Invasive insects—an underestimated cost to the world economy | | *Phys.org* | | |  |  |
|  |  |  |  |  |  | Global costs of invasive insects | | *DNA barcoding* | | |  |  |
|  |  |  |  |  |  | Flexible Reproduction ‘Mite’ Explain Invasion Success | | *Entomology today* | | |  |  |
|  |  |  |  |  |  | Invasive tawny crazy ants have an intense craving for calcium – with implications for their spread in the US | | *Environewsbits* | | |  |  |
| 7 | mammals | | Wang et al. (2023) | | PlumX | 李义明研究团队揭示外来哺乳动物入侵的全球货币成本 | | *ScienceNet.cn* | | | 1 (1) | - |
| 8 | terrestrial invertebrates | | Renault et al. (2022) | | PlumX | - | | - | | | - | 18 |
|  |  | |  | |  |  | |  | | |  |  |
| **conceptual** | | | | | | | | | | | | |
| **no.** | **category** | | **citation** | | **Altmetric (or PlumX)** | **news story examples** | | **associated outlet(s)** | | | **news stories (outlets)** | **tweets (users)** |
| 1 | 100 worst | | Cuthbert et al. (2022a) | | 155 | - | | - | | | - | 235 (215) |
| 2 | canals | | Balzani et al. (2022) | | 6 | - | | - | | | - | 10 |
| 3 | cost dynamics | | Haubrock et al. (2022b) | | PlumX | Geographic and taxonomic trends of rising biological invasion costs | | *DocWireNews* | | | 1 (1) | 1 |
| 4 | inaction costs | | Ahmed et al. (2022b) | | 20 | - | | - | | | - | 42 (33) |
| 5 | modelling damage costs | | Ahmed et al. (2022a) | | 12 | - | | - | | | - | 22 (20) |
| 6 | nature of costs | | Vaissière et al. (2022) | | 13 | - | | - | | | - | 25 (21) |
| 7 | species with benefits + costs | | Kourantidou et al. (2022a) | | 16 | - | | - | | | - | 37 (28) |
|  |  | |  | |  |  | |  | | |  |  |
| **habitat** | | | | | | | | | | | | |
| **no.** | **category** | | **citation** | | **Altmetric (or PlumX)** | **news story examples** | | **associated outlet(s)** | | | **news stories (outlets)** | **tweets (users)** |
| 1 | aquatic | | Cuthbert et al. (2021b) | | PlumX | University Of Florida Scientist Helps Perfect Tool To Reduce Invasive Species Worldwide | | *NewsBreak* | | | 3 | 130 |
|  |  |  |  |  |  | Aquatic invasive species cause billions of dollars in damage | | *Phys.org* | | |  |  |
|  |  |  |  |  |  | Aquatic invasive species cause damage worth billions of dollars | | *EurekAlert!* | | |  |  |
| 2 | protected areas | | Moodley et al. (2022) | | 34 | - | | - | | | - | 62 (54) |
|  |  | |  | |  |  | |  | | |  |  |
| **sectoral** | | | | | | | | | | | | |
| **no.** | **category** | | **citation** | | **Altmetric (or PlumX)** | **news story examples** | | **associated outlet(s)** | | | **news stories (outlets)** | **tweets (users)** |
| 1 | biosecurity | | Cuthbert et al. (2022b) | | PlumX | Invasive reptile and amphibian species are causing billions of dollars in damages globally | | *Phys.org, The Conversation, NewsBreak* | | | 4 (9) | 63 |
|  |  |  |  |  |  | Invasive species prevention ‘could save trillions’ | | *Eco-Business, SciDev.net, SciDev.Net: Asia & Pacific* | | |  |  |
|  |  |  |  |  |  | Rack of squirrel, anyone? The chefs putting invasive species on the menu | | *MSN UK, The Guardian: Environment News* | | |  |  |
|  |  |  |  |  |  | Capitol rioter who dressed as Jack Skellington on Jan. 6 will be donning prison garb for up to 8 years | | *Raw Story* | | |  |  |
| 2 | pathways | | Turbelin et al. (2022) | | 9 | - | | - | | | - | 18 (17) |
|  |  | |  | |  |  | |  | | |  |  |
| **general** | | | | | | | | | | | | |
| **no.** | **category** | | **citation** | | **Altmetric (or PlumX)** | **news story examples** | | **associated outlet(s)** | | | **news stories (outlets)** | **tweets (users)** |
| 1 | database | | Diagne et al. (2020) | | 296 | BEYOND LOCAL: Invasive species are causing billions of dollars in damages globally | | *TimminsToday.com* | | | 32 (21) | 217 (187) |
|  |  |  |  |  |  | Invasive reptile and amphibian species are causing billions of dollars in damages globally | | *Winnipeg Free Press, FuturesTradingCharts.com, Phys.org, Foregin Affairs New Zealand, Phys.org, Newsbreak, The Conversation* | | |  |  |
|  |  |  |  |  |  | Invasive species have cost UK at least £5bn since 1970s, study reveals | | *Google News, Yahoo! News, Newsbreak, The Guardian* | | |  |  |
|  |  |  |  |  |  | Attack of the alien invaders | | *COSMOS Magazine, Newsbreak, The Conversation* | | |  |  |
|  |  |  |  |  |  | Ecology: Global cost of biological invasions(Scientific Data) | | *Nature Asia* | | |  |  |
| 2 | global costs | | Diagne et al. (2021a) | | 1033 | Pests are destroying Canada’s trees — and a warming climate threatens to send more insects north | | *MorningNews* | | | 72 (61) | 869 (786) |
|  |  |  |  |  |  | Invasive species are costing the world billions every year | | *The Boar* | | |  |  |
|  |  |  |  |  |  | Pest plants and animals leave a frightening $1.7 trillion bill | | *BrazilNews.net, Newsbreak, The Conversation* | | |  |  |
|  |  |  |  |  |  | Attack of the alien invaders: Pest plants and animals leave a $1.7 trillion bill | | *Phys.org* | | |  |  |
|  |  |  |  |  |  | Invasive exotic species: how much are they costing us? | | *MarketScreener* | | |  |  |
| 3 | non-English sources | | Angulo et al. (2021b) | | PlumX | Pest plants and animals leave a frightening $1.7 trillion bill | | South Africa Today | | | 2 (3) | 501 |
|  |  |  |  |  |  | Attack of the alien invaders: Pest plants and animals leave a $1.7 trillion bill | | Phys.org, The Conversation | | |  |  |
|  |  | |  | |  |  | |  | | |  |  |
| **geographical** | | | | | | | | | | | | |
| **no.** | **category** | | **citation** | | **Altmetric (or PlumX)** | **news story examples** | | **associated outlet(s)** | | | **news stories (outlets)** | **tweets (users)** |
| ***continental*** | | | | | | | | | | | | |
| 1 | Africa | | Diagne et al. (2021b) | | 6 | - | | - | | | - | 7 (7) |
| 2 | Asia | | Liu et al. (2021) | | 9 | - | | - | | | - | 14 |
| 3 | Europe | | Haubrock et al. (2021d) | | 64 | Fremmede arter koster milliarder | | *ABC Nyheter* | | | 3 (3) | 58 (44) |
|  |  |  |  |  |  | La costosa invasione delle specie aliene in Europa | | *Green Report* (*Italy*) | | |  |  |
|  |  |  |  |  |  | I danni delle specie invasive in Europa crescono di 10 volte ogni decennio | | *Innovazione* | | |  |  |
| 4 | North America | | Crystal-Ornelas et al. (2021) | | 41 | More than 400 Invasive Fish Dumped from Aquariums Found in Texas River | | *Smithsonian Magazine* | | | 1 (1) | 48 (39) |
| 5 | South and Central America | | Heringer et al. (2021) | | 11 | - | | - | | | - | 22 (19) |
| ***regional*** | | | | | | | | | | | | |
| 6 | Mediterranean | | Kourantidou et al. (2021) | | 24 | - | | - | | | - | 38 (30) |
| 7 | Nordic | | Kourantidou et al. (2022b) | | PlumX | - | | - | | | - | 2 |
| 8 | Southeast Asia | | Haubrock et al. (2021c) | | 10 | - | | - | | | - | 19 (15) |
| ***national*** | | | | | | | | | | | | |
| 9 | Argentina | | Duboscq-Carra et al. (2021) | | 28 | - | | - | | | - | 57 (43) |
| 10 | Australia | | Bradshaw et al. (2021a) | | 204 | To lock out foot-and-mouth disease, Australia must help its neighbour countries bolster their biosecurity | | *Phys.org, Futures TradingCharts.com, Yahoo! News, The Conversation, Newsbreak* | | | 27 (19) | 133 (65) |
|  |  |  |  |  |  | Rapid response team tackles invasive species | | *Australian Greens, Mirage News* | | |  |  |
|  |  |  |  |  |  | Fish robots put a scare into invasive species | | *COSMOS magazine* | | |  |  |
|  |  |  |  |  |  | Pest plants and animals cost Australia around $25 billion a year – and it will get worse | | *Outlook India, Devdiscourse, Mirage News, Newsbreak* | | |  |  |
|  |  |  |  |  |  | Ryegrass, fire ants and feral cats: major Australian study identifies costliest pests in past 60 years | | *MSN, The Guardian* | | |  |  |
| 11 | Brazil | | Adelino et al. (2021) | | 12 | - | | - | | | - | 7 (7) |
| 12 | Ecuador | | Ballesteros-Mejia et al. (2021) | | 6 | - | | - | | | - | 10 (9) |
| 13 | France | | Renault et al. (2021) | | 92 | A new species of flatworm in our gardens that comes from Asia: Humbertium covidum | | *Phys.org, Foreign Affairs New Zealand, Global Advisors, The Conversation* | | | 7 (6) | 66 (55) |
|  |  |  |  |  |  | Biodiversité : Pourquoi les « invasions biologiques » coûtent (vraiment) très cher à la France | | *MSN* | | |  |  |
|  |  |  |  |  |  | News story from 20minutes on Wednesday 15 September 2021 | | *20 Minutes* | | |  |  |
|  |  |  |  |  |  | Les invasions biologiques, un fardeau économique pour la France | | *The Conversation* | | |  |  |
| 14 | Germany | | Haubrock et al. (2021a) | | 6 | - | | - | | | - | 10 (9) |
| 15 | India | | Bang et al. (2022) | | 47 | Colonialism Changed the Way Plants Are Distributed Around the World: Study | | *The Wire* | | | 4 (4) | 34 (23) |
|  |  |  |  |  |  | Poorly planned plantation drives are helping invasive species bloom in India | | *Moneycontrol.com* | | |  |  |
|  |  |  |  |  |  | भारत में घुसपैठ कर गए जीव-जंतुओं से हो रहा लाखों-करोड़ों का नुकसान | | *Yourstory* | | |  |  |
|  |  |  |  |  |  | “Aliens” have cost the Indian economy $127 billion over 60 years | | *Quartz* | | |  |  |
| 16 | Italy | | Haubrock et al. (2021b) | | 7 | - | | - | | | - | 6 (6) |
| 17 | Japan | | Watari et al. (2021) | | 34 | - | | - | | | - | 56 (56) |
| 18 | Mexico | | Rico-Sánchez et al. (2021) | | 5 | - | | - | | | - | 9 (7) |
| 19 | New Zealand | | Bodey et al. (2022b) | | - | - | | - | | | - | 11 |
| 20 | Russia | | Kirichenko et al. (2021) | | 5 | - | | - | | | - | 9 (7) |
| 21 | Spain | | Angulo et al. (2021a) | | 28 | - | | - | | | - | 43 (34) |
| 22 | United Kingdom | | Cuthbert et al. (2021a) | | 110 | European Colonialism Has Had A Lasting Legacy On How Plants Are Distributed Around The World | | *Futures TradingCharts.com, Foreign Affairs New Zealand, Yahoo! News, The Conversation* | | | 15 (10) | 47 (35) |
|  |  |  |  |  |  | Specieswatch: run rabbit – why they are disappearing from the countryside | | *MSN, Newsbreak, Yahoo! News, The Guardian* | | |  |  |
|  |  |  |  |  |  | Fremmede arter koster milliarder | | *ABC Nyheter* | | |  |  |
|  |  |  |  |  |  | Invasive species cost UK economy over £5 billion over past 40-50 years | | *Envirotec Magazine, Google News, Yahoo! News, Newsbreak, The Guardian* | | |  |  |
| 23 | USA | | Fantle-Lepczyk et al. (2022) | | PlumX | Noble false widows: The tiny spiders taking a big bite out of British and Irish wildlife | | *Phys.org, The Conversation* | | | 7 | 64 |
|  |  |  |  |  |  | the tiny spiders taking a big bite out of British and Irish wildlife | | *United Kingdom KNews.MEDIA* | | |  |  |
|  |  |  |  |  |  | Rat killers in paradise: an eradication program remakes a tropical atoll | | *Mongabay* | | |  |  |
|  |  |  |  |  |  | Auburn University researcher co-authors study determining economic impact of invasive species in US exceeds $1.2 trillion | | *RocketNews, AgriMarketing* | | |  |  |
|  |  |  |  |  |  | Invasive Species Cost the US $21 Billion per Year, Study Finds | | *FocusOn Equipment Rentals* | | |  |  |

| **Table S4 Summary of the descriptors** *Type of cost merged* and *Management type* used in *InvaCost* database. The complete summary of the descriptive columns of *InvaCost* is available at: github.com/Farewe/invacost/blob/master/data-raw. | |
| --- | --- |
|  |  |
| **Type of cost merged** | **Categories of the Type of cost column reassigned** |
| Damage | Economic losses due to direct and/or indirect impacts of invaders, such as yield loss, health injury, land alteration, infrastructure damage, or income reduction. |
| Management | In a broad sense of management. When monetary resources are allocated to mitigate the spread or impacts of invaders, such as prevention, control, research, long-term management (*sensu stricto*), eradication. |
| Mixed | When costs included both ‘damage’ and ‘management’ components. |
| Unspecified | Every cost for which the nature of cost was not clearly defined. |
| **Management type** | **Management in a broad sense in relation to the moment of the invasive species introduction** |
| Pre-invasion management | Monetary investments for preventing successful invasions in an area — including quarantine or border inspection, risk analyses, biosecurity management, etc. |
| Post-invasion management | Money spent for species management in invaded areas - including control, eradication, containment. |
| Knowledge/funding | Money allocated to all actions and operations that could be of interest at all steps of management at pre- and post-invasion stages — including administration, communication, education, research, etc. |
| Mixed | When costs include at least (and without possibility to disentangle the specific proportion of) two of the previous categories. |
| Unspecified | Every cost for which the nature of cost was not clearly defined. |
| NA | Every entry that has partly or fully associated with damage costs was assigned. |

| **References** |
| --- |
| Adelino JRP, Heringer G, Diagne C, Courchamp F, Faria LDB, Zenni RD. 2021. The economic costs of biological invasions in Brazil: a first assessment. NeoBiota 67:349-374.  Ahmed DA, Hudgins EJ, Cuthbert RN, Haubrock PJ, Renault D, Bonnaud E, Diagne C, Courchamp F. 2022a. Modelling the damage costs of invasive alien species. Biological Invasions 24:1949-1972.  Ahmed DA, et al. 2022b. Managing biological invasions: the cost of inaction. Biological Invasions 24:1927-1946.  Angulo E, Ballesteros-Mejia L, Novoa A, Duboscq-Carra VG, Diagne C, Courchamp F. 2021a. Economic costs of invasive alien species in Spain. NeoBiota 67:267-297.  Angulo E, et al. 2021b. Non-English languages enrich scientific knowledge: the example of economic costs of biological invasions. Science of the Total Environment 775:144441.  Angulo E, et al. 2022. Economic costs of invasive alien ants worldwide. Biological Invasions 24:2041-2060.  Ballesteros-Mejia L, Angulo E, Diagne C, Cooke B, Nuñez MA, Courchamp F. 2021. Economic costs of biological invasions in Ecuador: the importance of the Galapagos Islands. NeoBiota 67:375-400.  Balzani P, Cuthbert RN, Briski E, Galil B, Castellanos-Galindo GA, Kouba A, Kourantidou M, Leung B, Soto I, Haubrock PJ. 2022. ﻿Knowledge needs in economic costs of invasive species facilitated by canalisation. NeoBiota 78:207-223.  Bang A, Cuthbert RN, Haubrock PJ, Fernandez RD, Moodley D, Diagne C, Turbelin AJ, Renault D, Dalu T, Courchamp F. 2022. Massive economic costs of biological invasions despite widespread knowledge gaps: a dual setback for India. Biological Invasions 24:2017-2039.  Bernery C, Leroy B, Diagne C, Courchamp F. 2021. 1 288 milliards de dollars : chiffrer les dégâts causés par les invasions biologiques pour enfin agir. The Conversation. theconversation.com/1-288-milliards-de-dollars-chiffrer-les-degats-causes-par-les-invasions-biologiques-pour-enfin-agir-158204.  Bodey TW, Angulo E, Bang A, Bellard C, Fantle-Lepczyk J, Lenzner B, Turbelin A, Watari Y, Courchamp F. 2022a. Economic costs of protecting islands from invasive alien species. Conservation Biology:e14034.  Bodey TW, Carter ZT, Haubrock PJ, Cuthbert RN, Welsh MJ, Diagne C, Courchamp F. 2022b. Building a synthesis of economic costs of biological invasions in New Zealand. PeerJ 10:e13580.  Bradshaw CJA, Hoskins A. 2021. Pest plants and animals cost Australia around $25 billion a year – and it will get worse. The Conversation. theconversation.com/pest-plants-and-animals-cost-australia-around-25-billion-a-year-and-it-will-get-worse-164969.  Bradshaw CJA, et al. 2021a. Detailed assessment of the reported economic costs of invasive species in Australia. NeoBiota 67:511-550.  Bradshaw CJA, Leroy B, Bellard C, Roiz D, Albert C, Fournier A, Barbet-Massin M, Salles J-M, Simard F, Courchamp F. 2016. Massive yet grossly underestimated global costs of invasive insects. Nature Communications 7:12986.  Bradshaw CJA, Leroy B, Bernery C, Diagne C, Courchamp F. 2021b. Attack of the alien invaders: pest plants and animals leave a frightening $1.7 trillion bill. The Conversation. theconversation.com/attack-of-the-alien-invaders-pest-plants-and-animals-leave-a-frightening-1-7-trillion-bill-158628.  Courchamp F. 2021. Les invasions biologiques, un fardeau économique pour la France. The Conversation. theconversation.com/les-invasions-biologiques-un-fardeau-economique-pour-la-france-165119.  Crystal-Ornelas R, et al. 2021. Economic costs of biological invasions within North America. NeoBiota 67:485-510.  Cuthbert RN. 2022. A world of invaders. The Environment, Chartered Institute of Water and Environmental Management. ciwem.org/the-environment/a-world-of-invaders.  Cuthbert RN, Bartlett AC, Turbelin AJ, Haubrock PJ, Diagne C, Pattison Z, Courchamp F, Catford JA. 2021a. Economic costs of biological invasions in the United Kingdom. NeoBiota 67:299-328.  Cuthbert RN, Diagne C, Haubrock PJ, Turbelin AJ, Courchamp F. 2022a. Are the “100 of the world’s worst” invasive species also the costliest? Biological Invasions 24:1895-1904.  Cuthbert RN, et al. 2022b. Biological invasion costs reveal insufficient proactive management worldwide. Science of the Total Environment 819:153404.  Cuthbert RN, et al. 2021b. Global economic costs of aquatic invasive alien species. Science of the Total Environment 775:145238.  Diagne C, Leroy B, Gozlan RE, Vaissière AC, Assailly C, Nuninger L, Roiz D, Jourdain F, Jarić I, Courchamp F. 2020. *InvaCost*, a public database of the economic costs of biological invasions worldwide. Scientific Data 7:277.  Diagne C, Leroy B, Vaissière A-C, Gozlan RE, Roiz D, Jarić I, Salles J-M, Bradshaw CJA, Courchamp F. 2021a. High and rising economic costs of biological invasions worldwide. Nature 592:571-576.  Diagne C, et al. 2021b. The economic costs of biological invasions in Africa: a growing but neglected threat? NeoBiota 67:11-51.  Duboscq-Carra VG, Fernandez RD, Haubrock PJ, Dimarco RD, Angulo E, Ballesteros-Mejia L, Diagne C, Courchamp F, Nuñez MA. 2021. Economic impact of invasive alien species in Argentina: a first national synthesis. NeoBiota 67:329-348.  Eco. 2021. Invasive Species Cost UK Economy Over £5 Billion Over Past 40-50 Years. Eco Magazine. ecomagazine.com/news/policy/invasive-species-cost-uk-economy-over-5-billion-over-past-40-50-years.  Elmore SA. 2018. The Altmetric attention score: what does it mean and why should I care? Toxicologic Pathology 46:252-255.  European Commission Directorate-General for Environment. 2022. Expenditure on preventing biological invasions is far below environmental damage costs. Science for Environment Policy. environment.ec.europa.eu/news/expenditure-preventing-biological-invasions-far-below-environmental-damage-costs-2022-10-05_en.  Fantle-Lepczyk JE, Haubrock PJ, Kramer AM, Cuthbert RN, Turbelin AJ, Crystal-Ornelas R, Diagne C, Courchamp F. 2022. Economic costs of biological invasions in the United States. Science of the Total Environment 806:151318.  Fernandez RD, Haubrock PJ, Cuthbert R, Heringer G, Kourantidou M, Hudgins EJ, Angulo E, Diagne CA, Courchamp F, Nuñez MA. 2022. Underexplored and growing economic costs of invasive alien trees. SSRN 10.2139/ssrn.4196468.  Haubrock PJ, et al. 2022a. Knowledge gaps in economic costs of invasive alien fish worldwide. Science of the Total Environment 803:149875.  Haubrock PJ, Cuthbert RN, Hudgins EJ, Crystal-Ornelas R, Kourantidou M, Moodley D, Liu C, Turbelin AJ, Leroy B, Courchamp F. 2022b. Geographic and taxonomic trends of rising biological invasion costs. Science of the Total Environment 817:152948.  Haubrock PJ, Cuthbert RN, Ricciardi A, Diagne C, Courchamp F. 2022c. Economic costs of invasive bivalves in freshwater ecosystems. Diversity and Distributions 28:1010-1021.  Haubrock PJ, Cuthbert RN, Sundermann A, Diagne C, Golivets M, Courchamp F. 2021a. Economic costs of invasive species in Germany. NeoBiota 67:225-246.  Haubrock PJ, Cuthbert RN, Tricarico E, Diagne C, Courchamp F, Gozlan RE. 2021b. The recorded economic costs of alien invasive species in Italy. NeoBiota 67:247-266.  Haubrock PJ, Cuthbert RN, Yeo DCJ, Banerjee AK, Liu C, Diagne C, Courchamp F. 2021c. Biological invasions in Singapore and Southeast Asia: data gaps fail to mask potentially massive economic costs. NeoBiota 67:131-152.  Haubrock PJ, et al. 2021d. Economic costs of invasive alien species across Europe. NeoBiota 67:153-190.  Heringer G, Angulo E, Ballesteros-Mejia L, Capinha C, Courchamp F, Diagne C, Duboscq-Carra VG, Nuñez MA, Zenni RD. 2021. The economic costs of biological invasions in Central and South America: a first regional assessment. NeoBiota 67:401-426.  Jiang X, Zheng P, Soto I, Haubrock PJ, Chen J, Ji L. 2022. Global economic costs and knowledge gaps of invasive gastropods. Ecological Indicators 145:109614.  Kirichenko N, Haubrock PJ, Cuthbert RN, Akulov E, Karimova E, Shneider Y, Liu C, Angulo E, Diagne C, Courchamp F. 2021. Economic costs of biological invasions in terrestrial ecosystems in Russia. NeoBiota 67:103-130.  Kouba A, Oficialdegui FJ, Cuthbert RN, Kourantidou M, South J, Tricarico E, Gozlan RE, Courchamp F, Haubrock PJ. 2022. Identifying economic costs and knowledge gaps of invasive aquatic crustaceans. Science of the Total Environment 813:152325.  Kourantidou M, et al. 2021. Economic costs of invasive alien species in the Mediterranean basin. NeoBiota 67:427-458.  Kourantidou M, Haubrock PJ, Cuthbert RN, Bodey TW, Lenzner B, Gozlan RE, Nuñez MA, Salles J-M, Diagne C, Courchamp F. 2022a. Invasive alien species as simultaneous benefits and burdens: trends, stakeholder perceptions and management. Biological Invasions 24:1905-1926.  Kourantidou M, et al. 2022b. The economic costs, management and regulation of biological invasions in the Nordic countries. Journal of Environmental Management 324:116374.  Leroy B, Kramer AM, Vaissière A-C, Kourantidou M, Courchamp F, Diagne C. 2022. Analysing economic costs of invasive alien species with the invacost r package. Methods in Ecology and Evolution 13:1930-1937.  Liu C, et al. 2021. Economic costs of biological invasions in Asia. NeoBiota 67:53-78.  Macêdo RL, Franco ACS, Kozlowsky-Suzuki B, Mammola S, Dalu T, Rocha O. 2022. The global social-economic dimension of biological invasions by plankton: grossly underestimated costs but a rising concern for water quality benefits? Water Research 222:118918.  Moodley D, et al. 2022. Surprisingly high economic costs of biological invasions in protected areas. Biological Invasions 24:1995-2016.  Pimentel D, Lach L, Zuniga R, Morrison D. 2000. Environmental and economic costs of nonindigenous species in the United States. BioScience 50:53-65.  Renault D, Angulo E, Cuthbert RN, Haubrock PJ, Capinha C, Bang A, Kramer AM, Courchamp F. 2022. The magnitude, diversity, and distribution of the economic costs of invasive terrestrial invertebrates worldwide. Science of the Total Environment 835:155391.  Renault D, Manfrini E, Leroy B, Diagne C, Ballesteros-Mejia L, Angulo E, Courchamp F. 2021. Biological invasions in France: alarming costs and even more alarming knowledge gaps. NeoBiota 67:191-224.  Rico-Sánchez AE, Haubrock PJ, Cuthbert RN, Angulo E, Ballesteros-Mejia L, López-López E, Duboscq-Carra VG, Nuñez MA, Diagne C, Courchamp F. 2021. Economic costs of invasive alien species in Mexico. NeoBiota 67:459-483.  Soto I, Cuthbert RN, Kouba A, Capinha C, Turbelin A, Hudgins EJ, Diagne C, Courchamp F, Haubrock PJ. 2022. Global economic costs of herpetofauna invasions. Scientific Reports 12:10829.  Soto I, Hudgins E. 2022. Invasive reptile and amphibian species are causing billions of dollars in damages globally. The Conversation. theconversation.com/invasive-reptile-and-amphibian-species-are-causing-billions-of-dollars-in-damages-globally-188680.  Turbelin AJ, Cuthbert RN, Essl F, Haubrock PJ, Ricciardi A, Courchamp F. 2023a. Biological invasions are as costly as natural hazards. Perspectives in Ecology and Conservation 10.1016/j.pecon.2023.03.002.  Turbelin AJ, et al. 2022. Introduction pathways of economically costly invasive alien species. Biological Invasions 24:2061-2079.  Turbelin AJ, Hudgins EJ, Catford JA, Cuthbert RN, Kourantidou M, Roiz D, Courchamp F. 2023b. Biological invasions as burdens to primary economic sectors. Research Square 10.21203/rs.3.rs-2444595/v1.  Vaissière A-C, Courtois P, Courchamp F, Kourantidou M, Diagne C, Essl F, Kirichenko N, Welsh M, Salles J-M. 2022. The nature of economic costs of biological invasions. Biological Invasions 24:2081-2101.  Wang S, Deng T, Zhang J, Li Y. 2023. Global economic costs of mammal invasions. Science of the Total Environment 857:159479.  Watari Y, Komine H, Angulo E, Diagne C, Ballesteros-Mejia L, Courchamp F. 2021. First synthesis of the economic costs of biological invasions in Japan. NeoBiota 67:79-101.  World Fishing and Aquaculture. 2021. Invaders in our waters: at what cost? World Fishing and Aquaculture. worldfishing.net/invaders-in-our-waters-at-what-cost/1405302.article. |
